# A Randomised Trial of Subcutaneous Intermittent Interleukin-2 without Antiretroviral Therapy in HIV-Infected Patients: The UK–Vanguard Study

**DOI:** 10.1371/journal.pctr.0010003

**Published:** 2006-05-19

**Authors:** Mike Youle, Sean Emery, Martin Fisher, Mark Nelson, Lisa Fosdick, George Janossy, Clive Loveday, Ann Sullivan, Christian Herzmann, Handan Wand, Richard T Davey, Margaret A Johnson, Jorge A Tavel, H. Clifford Lane

**Affiliations:** 1 Royal Free Centre for HIV Medicine, London, United Kingdom; 2 National Centre in HIV Epidemiology and Clinical Research, University of New South Wales, Sydney, Australia; 3 Brighton Healthcare Trust, Brighton, United Kingdom; 4 Kobler Centre, London, United Kingdom; 5 Division of Biostatistics, University of Minnesota, Minneapolis, United States of America; 6 National Institute of Allergy and Infectious Disease, National Institutes of Health, Bethesda, Maryland, United States of America

## Abstract

**Objective:**

The objective of the trial was to evaluate in a pilot setting the safety and efficacy of interleukin-2 (IL-2) therapy when used without concomitant antiretroviral therapy as a treatment for HIV infection.

**Design and Setting:**

This was a multicentre randomised three-arm trial conducted between September 1998 and March 2001 at three clinical centres in the United Kingdom.

**Participants:**

Participants were 36 antiretroviral treatment naïve HIV-1-infected patients with baseline CD4 T lymphocyte counts of at least 350 cells/mm^3^.

**Interventions:**

Participants were randomly assigned to receive IL-2 at 15 million international units (MIU) per day (12 participants) or 9 MIU/day (12 participants) or no treatment (12 participants). IL-2 was administered by twice-daily subcutaneous injections for five consecutive days every 8 wk.

**Outcome Measures:**

Primary outcome was the change from baseline CD4 T lymphocyte count at 24 wk. Safety and plasma HIV RNA levels were also monitored every 4 wk through 24 wk. The two IL-2 dose groups were combined for the primary analysis.

**Results:**

Area under curve (AUC) for change in the mean CD4 T lymphocyte count through 24 wk was 129 cells/mm^3^ for those assigned IL-2 (both dose groups combined) and 13 cells/mm^3^ for control participants (95% CI for difference, 51.3–181.2 cells/mm^3^; *p* = 0.0009). Compared to the control group, significant increases in CD4 cell count were observed for both IL-2 dose groups: 104.2/mm^3^ (*p* = 0.008) and 128.4 cells/mm^3^ (*p* = 0.002) for the 4.5 and 7.5 MIU dose groups, respectively. There were no significant differences between the IL-2 (0.13 log_10_ copies/ml) and control (0.09 log_10_ copies/ml) groups for AUC of change in plasma HIV RNA over the 24-wk period of follow-up (95% CI for difference, −0.17 to 0.26; *p* = 0.70). Grade 4 and dose-limiting side effects were in keeping with those previously reported for IL-2 therapy.

**Conclusions:**

In participants with HIV infection and baseline CD4 T lymphocyte counts of at least 350 cells/mm^3^, intermittent subcutaneous IL-2 without concomitant antiretroviral therapy was well tolerated and produced significant increases in CD4 T lymphocyte counts and did not adversely affect plasma HIV RNA levels.

## Editorial Commentary

**Background:** There is very good trial evidence that combinations of antiretroviral drugs improve the prognosis of people with HIV infection. However, these drugs can have major side effects, and HIV can become resistant to them. The development of alternative treatments might allow antiretroviral use to be delayed in some people, thereby reducing toxicity and the emergence of resistant strains of HIV.

**What this trial shows:** The researchers studied 36 adult patients in the United Kingdom who were infected with HIV and who had not previously been prescribed antiretrovirals. Participants in the trial were given either one of two different dosage levels of IL-2 (a chemical messenger normally produced in the body by particular immune cells) by injection under the skin or no treatment at all. Patients given IL-2 at either dosage experienced an increase in levels of CD4 T lymphocytes, the type of immune cell depleted by HIV infection, as compared to patients receiving no treatment. The viral load in patients receiving IL-2 did not differ from that of patients in the control arm.

**Strengths and limitations:** The rationale for this trial—to delay administration of antiretroviral drugs—is sound, as is the experimental design. The results are limited, however, by the short follow-up of patients in the trial and by the lack of clinical endpoints (such as disease progression), so it is not possible to tell whether patients in the trial benefited directly from the IL-2 treatment.

**Contribution to the evidence:** The clinical effectiveness and safety of IL-2 together with antiretrovirals as a treatment for HIV infection is currently being evaluated in other, large-scale trials. To date, no other study has assessed the outcomes arising from administration of IL-2 alone. Data from the current trial will be useful in planning further research to examine whether IL-2 might help delay antiretrovirals in HIV-positive people.

## INTRODUCTION

The development of combination antiretroviral therapy for the treatment of HIV infection has produced a marked decline in AIDS and death, but enthusiasm for these treatments in patients with early stages of HIV infection has been tempered by long-term toxicity, such as lipodystrophy and lactic acidosis, difficulties with maintaining rigorous compliance, and the evolution of drug resistant HIV [1–5]. The use of these treatments for prolonged periods may not be achievable, and treatment guidelines continue to change [6–8]. For these reasons, the development of alternate therapies or treatment strategies continues. One such strategy is the administration of intermittent interleukin-2 (IL-2) to augment or preserve immune function [9–11].

IL-2 is a cytokine that in vivo is secreted by activated T lymphocytes. IL-2 regulates the proliferation, differentiation, and survival of lymphocytes, including CD4 T cells [12]. Increases in CD4 T lymphocyte count arising from the use of intermittent IL-2 in combination with antiretroviral therapy have been demonstrated consistently in a number of randomised clinical trials [13–21]. The use of recombinant IL-2 has been associated with transient rises of plasma HIV RNA levels in some patients [13–14]. However, no significant persistent increase in HIV RNA has been observed in IL-2 recipients when compared to controls treated with combination antiretroviral therapy [13–21]. In fact, a pooled analysis of long-term follow-up data from the first three randomised controlled trials of intermittent IL-2 suggested that IL-2 in combination with antiretroviral therapy produced larger decreases in viral load than antiretroviral therapy alone [22]. One randomised study similarly found that IL-2 in combination with antiretroviral therapy produced larger decreases in viral load than antiretroviral therapy alone [18], although these findings were not observed in other randomised studies of short duration [13–17,19–21].

The purpose of this randomised controlled pilot trial was to determine whether intermittent IL-2 therapy administered without concomitant antiretroviral therapy safely increased CD4 T lymphocyte counts. Ultimately, if this strategy were to be successful, it might lead to a delay in the time at which chronic antiretroviral therapy would need to be initiated. Further trials would be required from which to draw any definitive conclusions.

## METHODS

### Participants

Patients 18 y or older who had HIV-1 infection and CD4 T lymphocyte counts of at least 350 cells/mm^3^ at screening were eligible for enrollment if they had never received IL-2 or antiretroviral therapy. Additional eligibility criteria required that participants had no history of AIDS-defining illness and had received no corticosteroids, cytotoxic chemotherapy, or experimental cytotoxic therapy in the preceding 4 wk. Participants were required to have blood cell profiles and serum chemistry values within acceptable ranges. The study was approved by the National Institute of Allergy and Infectious Diseases' institutional review board and also by each site's research ethics committee. All participants provided written informed consent. An independent data and safety monitoring board reviewed safety and efficacy data on one occasion during the conduct of the trial. Patients were recruited and followed, and one of three participants' sites provided primary healthcare.

### Interventions

Participants were randomly assigned in equal proportions to intermittent subcutaneous injections of IL-2 at two dosage levels (4.5 million international units [MIU] or 7.5 MIU twice daily for five consecutive days every 8 wk) or no treatment. The study was not placebo controlled, as the constitutional side effects of IL-2 make blinding impractical. IL-2 (aldesleukin [Proleukin], Chiron, Emeryville, California, United States) was administered either in a hospital clinic or through an outpatient department. Dose modifications in decrements of 1.5 MIU were allowed for the management of clinical or laboratory toxicities.

### Objectives

Our primary hypothesis was that intermittent cycles of IL-2 would result in significant increases in CD4 T lymphocyte counts relative to no therapy. Our secondary hypotheses were that there would be no significant increases in plasma HIV RNA between treatment groups and that IL-2 cycles would be safe and well tolerated, in keeping with the experience of earlier studies.

### Outcomes

Over the 24-wk study period, participants were evaluated monthly, with additional visits of the IL-2-treated participants at day 5 of each cycle. Clinical assessments involved physical examination, complete blood counts with differentials and platelet counts, serum chemistry profiles, T lymphocyte subset enumeration, and plasma HIV RNA quantitation.

Absolute CD4 and CD8 T lymphocyte counts were determined from 100 μl of fresh EDTA blood, by direct immunofluorescence using the Ortho-Trio method [23,24]. Particle-associated plasma HIV RNA concentrations were determined using a branch-chain DNA assay (Chiron) with a lower limit of detection at 50 copies/ml plasma [25,26]. Flow cytometry and HIV quantitation were performed at a single laboratory throughout the study.

A treatment failure was prospectively defined as any patient who experienced either at least a 1-log increase in plasma HIV RNA on two consecutive occasions more than 29 d apart in the absence of an intercurrent illness, a greater than 25% reduction from the baseline CD4 T lymphocyte count on two occasions more than 29 d apart in the absence of intercurrent illness, and/or initiation of antiretroviral therapy for any reason.

### Sample Size

Sample size was specified as 36 participants in order to provide 80% power to detect a difference between both IL-2 dose groups combined and the control group of 150 CD4 T lymphocytes/mm^3^ at a two-sided significance level of 5%. With this sample size, power was also 80% to detect a difference of 0.7-log copies of HIV RNA per cubic millimeter between treatment groups. These estimates were considered conservative because they did not consider the averaging of multiple follow-up measurements of CD4 and HIV RNA that were to be used for the primary analysis.

### Randomisation

Randomisation was performed through using a central randomisation office located at the University of Minnesota. Computer-generated randomisation lists were generated at this office using a blocking factor of 6. Allocation of patients was by facsimile request from participant sites to the University of Minnesota randomisation office.

### Statistical Methods

The primary end points of the study were area under the curve (AUC) for CD4 T lymphocyte count change from baseline and AUC for plasma HIV RNA change from baseline over the 24 wk of the study. The AUC estimates were standardised for the timing of the last measurement for each person [27]. Secondary end points included the comparative frequency of protocol-defined treatment failure, changes in percentage of CD4 T lymphocytes, the number and percentage of CD8 T lymphocytes, the CD4/CD8 T lymphocyte ratio, and safety data. Plasma HIV RNA data were log_10_ transformed prior to analyses.

Baseline was the average of three measurements made within 30 d of randomisation. Levels at 24 wk were the average of three measurements within 2 wk of one another. Follow-up levels at other weeks were based on a single reading.

Data for patients assigned IL-2 were analysed on an intention-to-treat basis; i.e., all follow-up measurements were included even if the patient was not taking IL-2. Data from one participant in the control group who initiated antiretroviral therapy are only included up to the time antiretroviral therapy commenced. As stated in the protocol, for the primary analyses, the combined data from the two IL-2 dose groups were compared with control participants. Pairwise comparisons between each of the randomly assigned treatment arms and the control group and with one another were also carried out. In addition to AUC analyses, longitudinal regression methods that take into account correlations within and between participants were used to estimate the average difference between treatment groups (IL-2 and control) over follow-up and to estimate the differences in CD4 T lymphocytes and plasma HIV RNA at each follow-up timepoint [28]. These analyses were carried out using the PROC Mixed procedure of the SAS Institute (Cary, North Carolina, United States). Other analyses were also carried out with the use of the SAS Version 8 software. All statistical tests were two-sided, with a *p*-value of <0.05, indicating statistical significance.

## RESULTS

### Patient Disposition, Recruitment, and Baseline Characteristics

**Figure 1:**
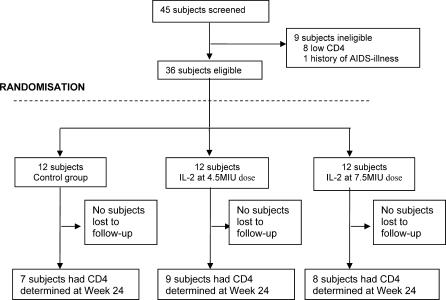
Flow Diagram of Study Design and Patient Disposition

A total of 45 patients were screened for participation. Disposition of the cohort over the entire period of follow-up is shown in Figure 1. Prior AIDS diagnoses (one participant) and low CD4 cell counts (eight participants) accounted for all ineligible screens. The remaining 36 participants were enrolled between September 1998 and August 1999. Of these, 12 were randomly assigned to each of the IL-2 treatment groups (giving a total of 24 IL-2 recipients) and 12 to the control group. No patients were lost to follow-up for the purposes of clinical assessment. However, five control patients and seven IL-2 recipients did not contribute laboratory data to the week 24 assessments. The baseline characteristics of the three groups were similar (Table 1).

**Table 1:**
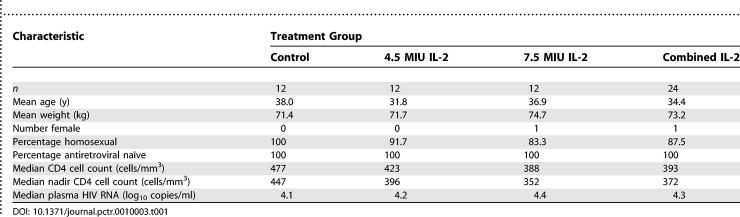
Demography and Baseline Characteristics

### Exposure to IL-2

The exposure of participants randomised to receive IL-2 is summarised in Table 2. During the 24-wk study period, most participants in either treatment group completed three treatments with IL-2. However, participants randomised to receive the 7.5 MIU dose reduced more frequently than those randomised to 4.5 MIU, and at week 24 the average unit dose of IL-2 was only 5.8 MIU. This is in contrast with the 4.2 MIU average unit dose for patients randomised to receive IL-2 at 4.5 MIU/dose.

**Table 2:**
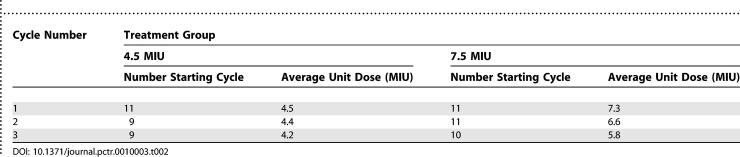
Exposure of Participants to IL-2, Shown by Number of Participants Commencing Each Cycle and Average Unit Dose in Each Cycle by Treatment Group

### Immunologic Measures

**Figure 2:**
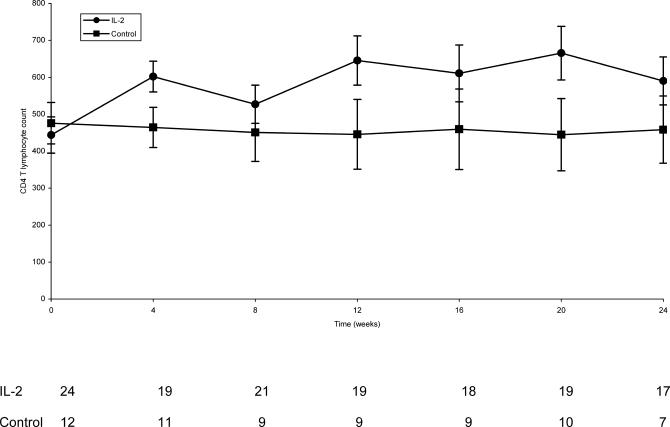
CD4^+^ Cell Count with 95% CI and Number of Patients with CD4 Cell Count Used in the Analysis

CD4 T lymphocyte counts over follow-up for each treatment group, based on longitudinal regression, are illustrated in Figure 2. At each follow-up visit except the 8-wk visit, CD4 T lymphocyte increases from baseline were significantly greater for those assigned IL-2 compared to control. At 24 wk, this difference was 132 cells/mm^3^ (*p* = 0.02). AUC for change in the mean CD4 T lymphocyte count through 24 wk was 129 cells/mm^3^ for those assigned IL-2 (both dose groups combined) and 13 cells/mm^3^ for control participants (95% CI for difference, 51.3–181.2 cells/mm^3^; *p* = 0.0009). As shown in Table 3, the AUC for change in CD4 T lymphocyte count from baseline was significantly greater for those assigned IL-2 (+129.4 cells/mm^3^) compared to control (+13.1 cells/mm^3^) (difference, 116.2 cells/mm^3^; 95% CI for difference, 51.3–181.2 cells/mm^3^). Longitudinal regression analysis yielded a similar difference between treatment groups (132.3 cells/mm^3^; *p* = 0.0001). Statistically significant differences were also observed for the pairwise comparisons of each IL-2 dose with control (*p* = 0.002 for 7.5 MIU versus control; *p* = 0.008 for 4.5 MIU versus control), but not for the comparison of IL-2 doses 4.5 MIU versus 7.5 MIU (*p* = 0.57). The numbers of CD8 T lymphocytes in each treatment group remained stable throughout the period of observation in each treatment group (unpublished data).

**Table 3:**
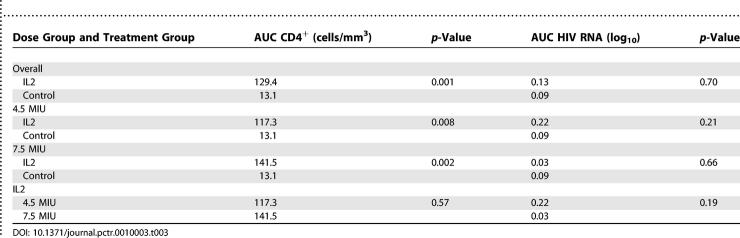
AUC for CD4 T Lymphocyte Count and HIV RNA Change from Baseline

### Virologic Measures

The AUC for change in plasma HIV RNA from baseline was similar for those assigned IL-2 (0.13 log_10_ copies/ml) and control (0.09 log_10_ copies/ml) (95% CI for difference, −0.17 to 0.26; *p* = 0.70) (Table 3). Similarly, there were no significant differences between treatment groups for mean changes in log_10_ HIV RNA at any timepoint in this parameter. Analyses based on longitudinal regression produced results similar to AUC (average difference, 0.11; *p* = 0.32).

Evaluation of plasma HIV RNA at the end of each 5-d cycle of IL-2 administration revealed a transient increase of at least 0.5 log_10_ HIV RNA in 32% of patients in cycle 1, 40% of patients in cycle 2, and 37% of patients in cycle 3. These increases ranged from 0.5 to 1.6 log_10_ HIV RNA. There were no statistically significant differences between the 4.5 MIU and 7.5 MIU dose groups (unpublished data).

### Toxicities and Safety Data

Following randomisation, one participant in each IL-2 dose group declined IL-2 therapy before receiving their first cycle, but their data have been included in the 24-wk analyses. One additional participant in the 4.5 MIU dose group permanently discontinued IL-2 after completing one cycle, citing personal reasons. Two additional participants in the 7.5 MIU dose group discontinued therapy, citing toxicity (one participant after completing two cycles), and multiple reasons, including toxicity and CD4 T lymphocyte count decrease (one participant after completing three cycles), as the reasons for discontinuation. No new side effects were encountered. The most numerous dose-limiting events were constitutional signs and symptoms, including fever and nausea. Five grade 4 events were reported during the trial as follows: diarrhea (4.5 MIU), pancreatitis (7.5 MIU), abdominal pain with hyperamylasemia (7.5 MIU), elevated alanine aminotransferase (4.5 MIU), and abdominal cramps with diarrhea (control). There were no deaths during follow-up.

### Protocol-Defined Treatment Failures and Clinical Disease Progression

On the basis of the protocol definition, three patients experienced treatment failure during the 24-wk follow-up period: two control participants (one commenced antiretroviral therapy at week 16 after being diagnosed with nonvisceral Kaposi's sarcoma and one had a decreased CD4 count); and one participant assigned 7.5 MIU IL-2 (who commenced antiretroviral therapy at week 24 after being diagnosed with visceral Kaposi's sarcoma).

## DISCUSSION

### Overall Evidence

Studies of intermittent administration of IL-2 in combination with antiretroviral therapy have demonstrated significant and sustained increases in CD4^+^ T cell count resulting from a preferential increase in CD4^+^ T cell survival and decreased cell turnover in the setting of decreased immune activation [29,30]. In this pilot study, CD4 T lymphocyte counts increased significantly in the IL-2 monotherapy arm, compared with the control group, at week 24. Importantly, these increases were not associated with sustained increases in HIV RNA load. Measurements of plasma HIV RNA in IL-2 recipients at the end of each 5-d cycle of IL-2 indicated that transient bursts of viremia were consistent with those observed in previous trials in which patients were treated with what would currently be regarded as suboptimal regimens of antiretroviral therapy [3–15]. Despite these transient bursts, there were no long-term changes in viral load.

The clinical significance of the increase in CD4 T lymphocytes that are produced under the influence of IL-2 therapy is uncertain and has led to the initiation of two large clinical endpoint studies (SILCAAT and ESPRIT [31]) to assess the clinical consequences of IL-2 in combination with antiretroviral therapy. SILCAAT and ESPRIT are sister studies, the former assessing HIV-infected participants with between 50 and 300 cells/mm^3^ and HIV RNA levels of <10,000 copies/ml, and the latter in participants with ≥300 cells/mm^3^ and no restriction on viral load.

Prior to ESPRIT, four Vanguard studies were conducted to address methodological and operational issues for studies of IL-2 therapy [19–21]. The pilot study reported here, the UK–Vanguard, was initiated to examine IL-2 treatment without antiretroviral medication. A striking feature of the data from this study relative to that from the others is a relatively blunted CD4 T lymphocyte count response. The mean increase in CD4 T lymphocyte count observed at 24 wk compared to control was 132 cells/mm^3^, considerably less than that observed in the other three Vanguard studies (an average increase of 328, 459, and 347 from baseline above those achieved by the control groups [19–21].

### Interpretation

The reasons for the clearly blunted response in the current trial are not known, but one possible explanation is that, in patients with ongoing uncontrolled virus replication, a larger proportion of the newly emerging CD4 T lymphocytes are eliminated. It is less likely that these differences in CD4 T lymphocyte count increases are due to the doses of IL-2 actually administered. In the other Vanguard studies, the average total doses of IL-2 given over the first three cycles to the 7.5 MIU arm (maximum of 225 MIU) were about 198–225 MIU, while in this study the average total dose of IL-2 given over the first three cycles to the 7.5 MIU arm was only minimally lower (192 MIU).

In this protocol we did not assess T-cell function. Neither did we choose to examine distinct subsets of CD4^+^ T lymphocytes that are believed to be of significance in the immunopathogenesis of HIV disease. In many other trials of subcutaneous IL-2, a consistent observation has been that the functionality and/or immunophenotype of T cells present prior to IL-2 administration remains following IL-2 treatment, although in some settings there appears to be a preferential expansion of naïve CD4^+^ T lymphocytes [29–30,32]. A role for IL-2 in restoring or perhaps inducing anti-HIV-specific immune responses has not been part of the hypothesis behind the therapeutic evaluation of IL-2 in our hands. In the current study we were encouraged by the absence of any within- or between-treatment group differences in HIV RNA plasma load. We infer from this observation that any active anti-HIV-specific immune responses were unaffected by the administration of IL-2 in this study.

### Generalisability

As observed in other trials, this study demonstrates that IL-2 is well tolerated at doses that produce significant increases in CD4 T lymphocyte counts. Toxicities occurred only during the intermittent cycles of IL-2, were mostly mild to moderate in severity, and were managed with a comprehensive approach that included dose modification and the use of medications to control signs and symptoms. Grade 4 events occurred once in four IL-2 recipients and once in a control patient. Across the 66 treatment cycles initiated in this trial, this number of grade 4 events indicates a prevalence of approximately 6% for events of this severity. No novel toxicities were observed.

While the sample size for this study was only 36 and not all patients provided data at the final timepoint for the primary measure of interest, the lower limit of the 95% CI for CD4 T lymphocyte (51 cells/mm^3^) and the upper limit for plasma HIV RNA difference from control (0.32 copies/ml) indicate that at least modest CD4 T lymphocyte increases are possible without adversely affecting viral load. Thus, these findings are sufficiently encouraging to plan other studies of intermittent monotherapy with IL-2 to study its potential for increasing or maintaining CD4 T cell counts and prolonging the time to initiation of antiretroviral therapy.

In summary, this pilot study demonstrated that intermittent IL-2 therapy alone can be used to safely and significantly improve CD4 T lymphocyte counts in HIV-infected individuals with baseline CD4 T lymphocyte counts >350 cells/mm^3^ with no detrimental effect on HIV replication as measured by plasma HIV RNA load. Ongoing studies are addressing the clinical consequences of these CD4 T lymphocyte rises and the significance of these findings in terms of current models used to describe the interplay between virus and host in the setting of HIV infection.

## SUPPORTING INFORMATION

CONSORT Checklist Click here for additional data file. (54 KB DOC)

Trial Protocol Click here for additional data file. (337 KB DOC)

## Abbreviations

AUC: area under curve
CI: confidence interval
IL-2: interleukin-2
MIU: million international units
