# Correction: A Randomised Trial of Subcutaneous Intermittent Interleukin-2 without Antiretroviral Therapy in HIV-Infected Patients: The UK-Vanguard Study

**DOI:** 10.1371/journal.pctr.0020023

**Published:** 2007-05-04

In *PLoS Clinical Trials,* volume 1, issue 1, doi: 10.1371/journal.pctr.0010003:

The following competing interest was declared by the author on submission, but due to an error the text was not included in the published version of the article: “Sean Emery receives research support from Abbott, Boehringer Ingelheim, Bristol-Myers Squibb, Chiron, GlaxoSmithKline, Merck Sharpe and Dohme, and Roche.”

