# Supplementary material for: A Randomised Trial of Subcutaneous Intermittent Interleukin-2 without Antiretroviral Therapy in HIV-Infected Patients: The UK–Vanguard Study
Source: PLoS Clin Trials. 2006 May 19;1(1):e3. doi: 10.1371/journal.pctr.0010003 (PMC1488892; doi:10.1371/journal.pctr.0010003)
Supplement: Trial Protocol [file pctr.0010003.sd002.doc]

**VERSION 1.2**

**A RANDOMIZED, OPEN LABEL PHASE II STUDY OF SUBCUTANEOUS**

**INTERLEUKIN-2 (PROLEUKIN^®^) ALONE VS. NO THERAPY IN PATIENTS WITH HIV INFECTION AND AT LEAST 350 CD4+ CELLS/MM^3^ WHO DO NOT WISH TO RECEIVE TREATMENT WITH ANTIRETROVIRAL THERAPY.**

SPONSORED BY:

Division of AIDS (DAIDS)

National Institutes of Health

Bethesda, Maryland U.S.A.

INVESTIGATIONAL SITES INVESTIGATORS

Royal Free Hospital Dr Michael Youle MB ChB

Pond Street Dr Margaret Johnson FRCP

London Tel:

NW3 2Q9 Fax:

Britain email:

Sussex House Dr Martin Fisher MRCP

Abbey Road Consultant Physician

Brighton Tel:

Sussex Fax:

BN2 1ES email:

Britain

Kobler Centre Dr Mark Nelson

Chelsea and Westminster Hospital Consultant Physician

369 Fulham Road Tel:

London Fax:

SW10 9TH email:

Britain

Dept Sexually Transmitted Diseases Professor Ian Weller BSc, MD, FRCP

University College London Med School Tel:

The Mortimer Market Centre Fax:

Mortimer Market email:

off Capper St

London

WC1E 6AU

Britain

CENTRAL VIROLOGY LAB INVESTIGATOR

CENTRAL IMMUNOLOGY LAB INVESTIGATOR

PROTOCOL MEDICAL MONITOR DATA MANAGEMENT

Jorge A. Tavel MD Sean Emery PhD

National Institutes of Health National Centre in HIV Epidemiology and

National Institute of Allergy and Clinical Research (NCHECR)

Infectious Diseases University of New South Wales

Bldg 10 Rm 8C402 376 Victoria St

9000 Rockville Pike Sydney, NSW 2010 Australia

Bethesda, MD 20892 USA (+612) 9332 4648 ph

(301)402 0980 x402 ph (+612) 9332-1837 fax

(301)402 1137 fax semery@nchecr.unsw.edu.au

jtavel@atlas.niaid.nih.gov

**A RANDOMIZED, OPEN LABEL PHASE II STUDY OF SUBCUTANEOUS**

**INTERLEUKIN-2 (PROLEUKIN^®^) ALONE VS. NO THERAPY IN PATIENTS WITH HIV INFECTION AND AT LEAST 350 CD4+ CELLS/MM^3^ WHO DO NOT WISH TO RECEIVE TREATMENT WITH ANTIRETROVIRAL THERAPY.**

**PRECIS:**

This protocol describes a randomized, phase II study of subcutaneous interleukin-2 (IL-2) therapy in HIV disease. The primary objectives of this protocol are to demonstrate the safety and tolerance of IL-2 in the absence of concomittant antiretrovirals, to demonstrate the immunological efficacy of IL-2 in the absence of antiretroviral therapy as defined by increases of CD4+ cell counts from baseline and to examine the effects of IL-2 on measures of plasma HIV-RNA as defined by changes from baseline. Approximately 36 patients with HIV infection, >350 CD4+ cells/mm^3^ and no history of opportunistic infection will be required to complete the trial. Individuals with no prior or current use of antiretroviral drugs will be able to participate in the study. Eligible patients will be randomly assigned to receive either no therapy or subcutaneous IL-2 at one of two doses every 12 hours for 5 days every 8 weeks. The two dose levels of IL-2 that will be studied are 4.5, and 7.5 million units (MIU) of IL-2. One group will receive 4.5 million units q12h x 5 days every eight weeks. An additinal group will receive 7.5 MIU q12h x 5 days every eight weeks. Following the completion of this study, patients randomized to receive IL-2 will be able to continue receiving IL-2 during follow-up or as a part of an additional protocol that will be based on the results of the current study. It is anticipated that this study will be a phase III trial involving a total of 3700 patients worldwide.

Table of contents

APPENDICES

I. SYNOPSIS

II. INTRODUCTION

III. RATIONALE

IV. OBJECTIVES

V. STUDY DESIGN

VI. PATIENT SELECTION

VII. TREATMENT PROCEDURES

VIII. STUDY PROCEDURES

IX. STUDY WITHDRAWAL

X. TRIAL MANAGEMENT

XI. ADVERSE EVENTS AND MANAGEMENT

XII. INSTITUTIONAL REVIEW BOARD APPROVAL/ETHICS COMMITTEE ACCEPTANCE

XIII. INFORMED CONSENT

XIV. COMPENSATION, INSURANCE AND INDEMNITY

XV. PROTOCOL AMENDMENTS

XVI. DRUG ACCOUNTABILITY

XVII. LABORATORY REQUIREMENTS

XVIII. CASE REPORT FORMS

XIX. STUDY MONITORING

XX. RETENTION OF RECORDS

XXI. USE OF INFORMATION AND PUBLICATION

XXII. REFERENCES

# APPENDICES

A. KARNOFSKY PERFORMANCE STATUS

B. CDC 1993. CASE DIAGNOSES - AIDS

C. INSTRUCTIONS FOR RECONSTITUTION OF IL-2 FOR ADMINISTRATION

D. MODIFIED CPCRA TOXICITY TABLE FOR GRADING ADVERSE EVENTS

E. GUIDELINES FOR IL-2 DOSE REDUCTION

F. SYMPTOMATIC MEDICATIONS RECOMMENDED FOR MANAGEMENT OF IL-2 RELATED SIDE EFFECTS

G. FLOW CYTOMETRY PANELS FOR CLINICAL ASSESSMENT

H. PREPARATION/STORAGE GUIDELINES FOR HIV LOAD TESTING

I. MODIFIED CPCRA CRITERIA FOR CLINICAL HIV DISEASE PROGRESSION

J. 21 CFR 50 - REQUIREMENTS FOR INFORMED CONSENT

K. PRICE AND BREW STAGING SYSTEM FOR THE AIDS DEMENTIA COMPLEX

# I. SYNOPSIS

This protocol describes a randomized, phase II study of subcutaneous interleukin-2 (IL-2) therapy in HIV disease. There are three primary objectives:

1. to demonstrate the safety and tolerance of IL-2 in the absence of concomittant antiretrovirals.

2. to demonstrate the immunological efficacy of IL-2 in the absence of concomittant antiretroviral therapy as defined by increases of CD4+ cell counts from baseline.

3. To assess the effects of IL-2 in the absence of concomittant antiretroviral therapy on plasma HIV RNA levels as defined by changes from baseline.

Approximately 36 patients with HIV infection, > 350 CD4+ cells/mm^3^ and no history of opportunistic infection will be required to complete the trial. Individuals never previously treated with antiretrovirals will be eligible to participate. Eligible patients will be randomly assigned to receive one of two treatment regimens:

A. No therapy (n=12)

B. Subcutaneous IL-2 at one of 2 doses, q12h x 5 days every 8 weeks (n=24)

Two different dose levels of IL-2 will be studied. The first group will receive 4.5 million units (MIU) q12h x 5 days every eight weeks. The second group will receive 7.5 MIU q12h x 5 days every eight weeks. Following the completion of this study, continued treatment for all patients will be made available through an additional protocol that will be based upon the results of the current study. It is anticipated that this additional study will be a phase III trial involving a total of 3700 patients worldwide.

# II. INTRODUCTION

The clinical management of HIV disease relies upon the use of antiretroviral and antimicrobial chemotherapies. Antimicrobial agents are used to treat or suppress episodes of infectious disease arising from the profound immunodeficiency that characterizes HIV disease. Antiretroviral drugs, through inhibition of HIV replication, delay the progressive depletion and destruction of immunocompetence. Even at the time of maximal antiretroviral activity with these agents it is often possible to detect virus replication. Furthermore, HIV with reduced susceptibility to these agents can be isolated from patients who have received therapy for relatively short periods of time. Ultimately, in some patients continued use of antiretroviral chemotherapy no longer results in significant suppression of virus replication and destruction of the immune system continues unchecked. The purpose of the present study is to determine whether or not IL-2 could be an alternate strategy for treatment in patients with relatively early HIV disease who do not wish to commence antiretroviral therapy.

### A. INTERLEUKIN-2 IN HIV DISEASE

Interleukin-2 is a 15Kdalton, immunostimulatory protein produced in vivo by T-lymphocytes following activation by antigens. It is central to the appropriate responsiveness of the immune system challenged by invading pathogens. Its synthesis and release by CD4+ T-lymphocytes in particular, is of critical importance to the orchestrated events that follow detection of foreign antigen and lead to clearance of pathogen and resolution of disease. IL-2 results in the proliferation and maturation of both T- and B- lymphocytes (Smith, 1988). In vitro, exogenous IL-2 can enhance the NK and CTL activity of lymphocytes from patients with AIDS (Rook et al, 1985).

Immunotherapy of HIV disease with IL-2 has undergone considerable investigation in Phase I and II trials. These studies have defined the safety profile of IL-2 delivered in a number of different regimens as well as the virological and immunological changes resulting from treatment.

Initial Phase I studies were conducted in AIDS patients with KaposiÕs sarcoma. Purified, native IL-2 was administered by continuous intravenous infusion (CIV) for 5 days a week for four consecutive weeks at doses ranging from 250 to 250,000 units per day (Lane et al, 1984). In a second group of 21 patients recombinant IL-2 was administered by CIV for 5 days every eight weeks at doses ranging from 1,500 Units to 12.5 MIU per day (Murphy et al, 1988). In these studies toxicities were minimal and included hepatic dysfunction, febrile illness, eosinophilia, mild prolongation of PTT and proteinuria. There was an increase in the incidence of bacterial infections compared with patients receiving γ-interferon. Although treatment did not result in consistent clinical responses in patients with late-stage HIV disease, there was a consistent increase (up to five-fold) in circulating lymphocyte counts.

Additional research undertaken at the National Institutes of Health demonstrated that administration of CIV interleukin-2 at 12-18 MIU/day for 5 days every eight weeks in combination with antiretroviral therapy in patients with more than 200 CD4+ cells/mm^3^ prior to commencing treatment resulted in significant increases in CD4+ cell counts for up to 4 years (Kovacs et al, 1995a). In 6 of 10 such patients, the magnitude of the CD4+ cell increase was 50% or greater. In the 6 patients with 100-200 CD4+ cells/mm^3^ before therapy, more frequent dose reductions were required and only two individuals demonstrated more than a 50% increase in CD4+ cell count. In patients with fewer than 100 CD4+ cell/mm^3^, none experienced a significant increase in CD4+ cell count after therapy. Long-term follow-up of these 10 patients is shown in Figure 1.

In many patients a burst of HIV viremia was observed at the completion of each treatment cycle. Changes in HIV virus load (as measured by branched chain DNA analysis) over the entire study period were consistent with immunological changes. Patients with increased CD4+ cell counts had stable virus load during and after therapy, patients in whom no demonstrable increases in CD4+ cell number were observed had increases in virus load over time.

Additional immunological responses to therapy that were noted in this study included reductions in expression of HLA-DR on CD8+ T-lymphocytes, increases in the expression of CD25 on CD4+ T-lymphocytes and Vβ-subset data indicating that the CD4+ cell expansion was polyclonal.

Figure 1.

Kovacs and colleagues (1995b) have recently completed a randomized, controlled trial of CIV IL-2 in patients with HIV infection and more than 200 CD4+ cells/mm^3^. IL-2 was administered for 5 days every 8 weeks for 10 months. Recipients of antiretroviral therapy alone (n=29) demonstrated a mean reduction in CD4+ cell count of approximately 5 cells mm^3^/month over 14 months of study (Figure 2).

Figure 2.

Over the same time period, recipients of CIV IL-2 (n=30) experienced a mean increase in CD4+ cell count of approximately 37 cells mm^3^/month (p<0.001). During the study HIV virus load was determined at monthly intervals (Figures 3a and 3b). At the end of the study, the levels of virus in recipients of IL-2 were not different from baseline levels nor were they different from levels in patients receiving antiretroviral therapy alone.

Figure 3a.

Figure3b.

Administration of IL-2 by continuous intravenous infusion results in a number of side effects, some of which require dose modification for subsequent treatment cycles. Dose-limiting toxicities in patients with more than 200 CD4+ cells/mm^3^ include, elevations of serum creatinine levels, elevations in liver function tests, neutropenia, thrombocytopenia, diarrhea and vomiting as well as a number of severe subjective symptoms including myalgias, arthralgias, fatigue/malaise, nausea and fever. Less frequently patients experience considerable fluid retention, acalculus cholecystitis or rashes. The use of IL-2has also been associated with autoimmune thyroid disease, the exacerbation of psoriasis and worsening of glucose intolerance in a patient with pre-existing AODM.

Of particular importance, nearly all IL-2 toxicities resolve very rapidly upon completion of the treatment cycle (3-5 days after). This reflects the short half life of the agent.

One death has been reported in a patient receiving IL-2 by continuous intravenous infusion, occurring some 10 days after receiving his first cycle of immunotherapy. This patient was subsequently found to have also received trichosanthin (Compound Q) for a third time by intravenous infusion two days prior to death; an autopsy was not performed, and the cause of death is not known.

More recently, research conducted in Australia has confirmed the increments in CD4+ cell count that result from the administration of IL-2 by intravenous infusion (Figure 4). This study also demonstrated that these infusions could be administered on an outpatient basis. In addition, no sustained increments in HIV load were demonstrated in recipients of IL-2, again confirming an earlier observation.

Figure 4.

A third randomized trial, conducted at five investigational sites in the USA and sponsored by Chiron was designed to compare the relative efficacy of 0, 3, 4 or 5 day infusions of IL-2 every eight weeks for 6 cycles in a cohort of patients with HIV-infection and CD4+ cell counts in the range 100-300 cells/mm^3^. As a group the IL-2 treated patients showed a significant increase in CD4+ cell counts compared to controls patients (Figure 5). In common with all other randomized trials of IL-2 therapy this increase in CD4+ cell count occurred in the absence of increases in virus load.

Figure 5.

In total therefore, some 157 patients have been enrolled in randomized comparisons of antiretroviral therapy versus antiretrovirals plus 5-day CIV IL-2. In the period of time during which patients continued to receive randomly allocated study medication, there was a consistent significant increase in CD4+ cell counts for patients receiving the combination. Longer term follow-up of these patients is compromised by the fact that in all trials, patients were allowed to receive open label IL-2 after 6 cycles. Nonetheless, as demonstrated in Table 1 the group of patients initially randomized to receive 5-day CIV IL-2 in combination with antiretrovirals has experienced fewer opportunistic infections than patients initially assigned to receive antiretroviral therapy alone. In addition all of the opportunistic infections have occurred at CD4+ cell counts that are expected for the natural history of HIV disease.

### B. SUBCUTANEOUS (SC) IL-2

Despite the ability to administer CIV IL-2 safely to HIV-infected individuals, it is likely that such a regimen will be inappropriate for widespread clinical use. A less intrusive regimen of IL-2 would afford greater scope for clinical utility. The administration of IL-2 by subcutaneous injection has been evaluated in a number of clinical trials. One study, originally enrolling 18 patients and since amended to recruit a further 48 subjects with CD4+ cell counts >500 cells/mm^3^ is ongoing. The maximal tolerated dose of SC IL-2 was 15 MIU/day administered for five days every 8 weeks. Toxicities were similar, although generally less severe, to those observed with administration of CIV IL-2. Most patients also experienced local reactions characterized by erythema, tenderness and occasional nodularity. In common with observations derived from studies of CIV IL-2 in HIV disease an analysis of data from the original 18 patients has revealed the following trends: patient tolerance is directly related to HIV disease stage, higher baseline CD4+ counts are predictive of greater CD4+ increases in response to therapy and a favorable CD4+ response is generally disclosed within the first three treatment cycles (Figure 6).

Figure 6.

**Table 1. Clinical events and CD4+ cell count occurring in patients from three randomized trials of CIV IL-2.**

| **CLINICAL EVENTS** | **CD4+ CELL COUNT AT DIAGNOSIS** |
| --- | --- |
| **CONTROL PATIENTS** |  |
| MAC | 40, 255 |
| Toxoplasmosis | 74, 160 |
| E. Candidiasis | 135 |
| PML | 144 |
| Histoplasmosis | 21 |
| CMV | 19, 40, 111 |
| Pneumocystis carinii pneumonia | 11, 12 |
| HIV encephalopathy | 18, 50 |
|  |  |
| **CIV IL-2 (5 day infusion) PATIENTS** |  |
| Cryptosporidiosis | 202 |
| KaposiÕs sarcoma | 131, 82, 302 |
| MAC | 51 |
| Cryptococcal meningitis | 10 |

Once-daily SC dosing administered on days 1-5 and 8-12 of a 4-week cycle for 13 cycles, has been evaluated in a Phase I trial in San Francisco. Flu-like toxicities were marked during the second week of dosing and the MTD was 6 MIU/day. Substantial CD4+ cell count increases were seen in some patients. A total of 18 patients with CD4+ counts 200-500 cells/mm^3^ were enrolled in this trial. Seven patients discontinued early, four are currently still on study. Of the seven patients who completed therapy, 3 had >25% CD4+ increase based on pre-therapy and post-therapy CD4 counts.

Toxicities noted on this trial were relatively mild and consisted mostly of flu-like symptoms and injection-site reactions.

Twice daily SC dosing for 5 days of a four-week cycle has been evaluated in a five-arm Phase II trial conducted by community-based practitioners and sponsored by Applied Immune Sciences (AIS, Santa Clara, California). Patients in this trial were randomized to receive either 2MIU m^2^/dose (low dose) or 4 MIU m^2^/dose (high dose), twice daily with or without ex vivo expanded CD8+ T-lymphocytes for a total of 5 cycles (Deresinski et al, 1995). A total of 23 patients received IL-2 alone under this protocol. The study was controlled with a treatment arm of antiretroviral therapy alone. Toxicities from this trial were relatively mild and related to flu-like symptoms common to nearly all IL-2 regimens. One asplenic patient died following pneumococcal sepsis one week after completion of therapy. The contribution of IL-2 to this even is not known. Transient increases in HIV virus load were noted at day 5 of therapy (2-3 fold increases), although upon completion of study, levels of plasma HIV-RNA were not significantly greater than baseline levels. Recipients of low-dose IL-2 had a mean increase in CD4+ cell count of 34% (range -27 to 93) and recipients of high dose IL-2 had a mean increase of 38% (range -28 to 112). The absence of a dose response is thought to reflect the extent of dose reductions undertaken in the high-dose recipients. Increments in CD4+ cell count were seen in patients with CD4+ cell counts both above and below 350 cells/mm^3^ suggesting that a subcutaneous regimen might be applicable for patients with more advanced HIV disease.

### C. CONCLUSION

A total of three randomized phase II trials have been conducted that include as comparators both an antiretroviral therapy arm and a 5-day every 8 weeks CIV IL-2 treatment arm. These have yielded consistent data including significant increases in CD4+ cell count in the absence of sustained increases of virus load. A phase III clinical efficacy trial is the next step in determining the true clinical benefit of interleukin-2 therapy in HIV disease. Such a trial will recruit a large cohort of HIV infected patients with early HIV disease in a multinational collaboration. As a prelude to this undertaking a series of phase II studies will be undertaken to support the establishment of a phase III program in settings where IL-2 has not been used or to address specific questions of clinical importance related to the design of such a clinical endpoint trial.

It has also been proposed treatment with IL-2 therapy in the absence of concomittant antiretroviral drugs might be one strategy for the clinical management of patients with early HIV disease. There are no clinical data to determine the validity of this approach. This protocol will generate data on the use of IL-2 in patients with early HIV disease who do not wish to commence regimens of antiretroviral therapy.

# III. RATIONALE

Interleukin-2 administered subcutaneously results in increases in CD4+ cell count that might impact upon HIV disease progression. A phase III trial, recruiting large numbers of HIV positive patients is the next step in the development process. The effects of SC IL-2 in patients with HIV disease in the absence of antiretroviral therapy are not defined. To develop appropriate clinical experience with this approach, this phase II trial will allow administration of IL-2 without antiretroviral therapy to a small number of patients in sites wishing to participate in the phase III protocol.

# IV. OBJECTIVES

This study has three primary objectives: to demonstrate the safety and tolerability of subcutaneously administered IL-2 (at escalating doses delivered for 5 days every eight weeks) in the absence of concomittant antiretrovirals and to demonstrate the immunological efficacy (as demonstrated by increases in CD4+ cell count) and effects upon plasma HIV-RNA levels of SC IL-2 therapy relative to patients treated with no therapy.

Secondary objectives will also be addressed. These will include, assessments of infrastructure requirements, rate of patient recruitment, quality of clinical data and compliance with protocol.

# V. STUDY DESIGN

### A. OVERVIEW

This is a phase II open label study of escalating doses of subcutaneous (SC) IL-2 alone versus no therapy in patients with HIV infection and a CD4+ lymphocyte count of at least 350 cells/mm^3^. This protocol is intended to define the safety and tolerability, immunological superiority and virological neutrality of IL-2 immunotherapy over no therapy.

There will be 3 treatment arms (1 control; 2 IL-2). Eligible patients will be randomized to receive no therapy or IL-2 therapy with two discreet doses of SC IL-2 (delivered for five days every eight weeks).

A total of 36 patients will be required to complete a minimum of three cycles of treatment. Of these patients, 12 will be allocated to receive no therapy and 24 patients will receive SC IL-2. A cycle is defined as 8 calendar weeks to include as necessary one five day period of SC IL-2 therapy.

Dosing with SC IL-2 will commence at doses of 4.5 MIU or 7.5 MIU twice daily (q12h) for 5 days every eight weeks. No patient will be treated with more than 7.5 MIU q12h x 5 days every eight weeks and no patient will commence a treatment cycle at a dose of less than 1.5 MIU q12h x 5 days every eight weeks.

# VI. PATIENT SELECTION

### A. Patient population

A total of 36 eligible patients will be required to complete this protocol. Broadly, eligible patients will be asymptomatic HIV infected individuals with a CD4+ cell count of at least 350 cells/mm^3^.

### B. Inclusion criteria

Patients must fulfill all of the following inclusion criteria to be eligible for therapy,

a) documented HIV-1 infection (positive HIV-ELISA and confirmatory test by one of several methods).

b) 18 years old or greater.

c) a single CD4+ cell count of at least 350 cells/mm^3^ within 30 days prior to randomisation (to be conducted by central laboratory -Appendix G).

d) the following clinical laboratories must be observed within 30 days of randomisation:

i. serum AST < 4.9 times upper limit of normal range (ULN) for the study site

ii. serum bilirubin < 2 x ULN

iii serum creatinine < 2.0 mg/dl

iv granulocyte count > 1000 cells/ mm^3^

v. hemoglobin > 11.0 g/dl

vi. platelet count > 50000 cells/mm^3^

e) a Karnofsky Performance Status > 80 (appendix A).

f) women of childbearing potential must provide a negative serum or urine pregnancy test within 14 days prior to randomization.

g) men and women should employ best available barrier contraception at all times.

h) patients should provide written informed consent that conforms to local, national and international requirements for the protection of human subjects in medical research.

### C. Exclusion criteria

a) prior IL-2 therapy.

b) prior or current use of any approved or experimental antiretroviral drug.

c) concurrent malignancy other than mucocutaneous Kaposi sarcoma or malignancy treated within the past 5 years.

d) any concurrent or history of AIDS defining illness (CPCRA appendix B).

e) use of systemic corticosteroids, chemotherapy or experimental cytotoxic drugs within four weeks prior to study therapy.

f) use of any agent approved or experimental with clinically significant immunomodulatory effects.

g) any CNS abnormality that requires treatment with anti-seizure medication.

h) patients with current or historical CrohnÕs disease, psoriasis or other autoimmune/inflammatory diseases with potentially life threatening complications.

i) pregnant or lactating women.

j) occasional or habitual use of recreational drugs/alcohol that in the opinion of the principal investigator would affect patient safety and/or compliance.

k) patients with any serious psychiatric, medical and/or cognitive disturbance or illness that in the opinion of the principal investigator may affect safety, compliance or ability to provide written informed consent.

# VII. TREATMENT PROCEDURES

Treatment must only be administered by one of the institutions identified as an investigational site in this protocol.

### A. Product description

Information on formulation, toxicities, stability and storage of interleukin-2 (aldesleukin, Proleukin^®^) can be found in the Investigators Brochure.

Refer to Appendix C for information on reconstitution of interleukin-2.

Information on formulation, toxicities, dosing, stability and storage of antiretroviral drugs can be found on the package inserts for licensed therapies as provided.

### B. General considerations

Patients will be randomly assigned to receive trial medication in accordance with the schedule summarized in Table 2.

**Table 2. Randomization schedule and treatment assignments**

| TREATMENT ARM | RECRUITMENT (n) | STUDY DRUGS |
| --- | --- | --- |
| Control Group | 12 | no therapy |
| SC IL-2 | 12  12 | 4.5 MIU bid  7.5 MIU bid |

IL-2 will be delivered by twice daily subcutaneous injection for 5 days every 8 weeks. A cycle of therapy is defined as five days of IL-2 plus a further seven weeks.

### C. Dose modification

### 1. IL-2 Dose Modifications for Management of Toxicities

Adverse events will be graded according to the modified DAIDS/CPCRA Toxicity Table (Appendix D) used in association with the patientÕs clinical presentation. adverse events which may be dose-limiting are delineated by shaded boxes. The decision to dose-modify will be based on the clinical judgment of the principal investigator in discussion, as appropriate, with other investigators and/or the IND Sponsors. Patients who do not meet objective criteria for a dose-limiting adverse event but who find the side-effects intolerable, may be dose-modified according to the schedules described below.

Adverse events which in the InvestigatorÕs opinion are not IL-2 related but are delineated as dose-limiting may be discussed with the IND Sponsors. All adverse events must be reported in the case report forms regardless of the InvestigatorÕs assessment of their relationship to the drug.

The minimum dose that can be delivered under this protocol is 1.5 MIU q12h. The maximum dose will be 7.5 MIU q12h.

Recipients of IL-2 who develop toxicities will be managed as medically appropriate for the signs and symptoms of the toxicity. If the event or events are felt, in the opinion of the principal investigator to be dose-limiting, that cycle of therapy will be held until:

1. the DLT is no longer considered dose-limiting clinically, and

2. Grade 4 toxicities require that study drug be held until the toxicity decreases in severity to < Grade 2. Those toxicities which may require that study drug be held before Grade 4 occurs are shown shaded in Appendix D; IL-2 dose reduction guidelines for these shaded toxicities are given in Appendix E.

For the remainder of a cycle in which a DLT occurs, the patient may have their dose reduced by 1.5 MIU/dose. Dosing will continue to be delivered on a q12h schedule. Patients who require more than two dose modifications in a cycle of therapy will have that cycle of treatment terminated. Thus patients may be dose reduced to 6.0 MIU/dose and then to 4.5 MIU/dose during their first cycle of treatment if that cycle had commenced at 7.5 MIU/dose.

Patients who, during a cycle of therapy require dose modifications will have the subsequent cycles initiated at a dose below the level that gave rise to the dose limiting toxicity during the previous cycle. Subsequent dose reductions will occur, as necessary, in decrements of 1.5 MIU/dose delivered on a q12h schedule. Accordingly, patients can be dose-reduced in a step wise fashion from 7.5 MIU/dose to 4.5 MIU/dose to 1.5 MIU/dose. Patients who do not tolerate 1.5 MIU/dose will have IL-2 stopped.

The rationale for dose modification will be noted clearly in the case report forms. The maximum duration of dosing will be 5 days and will not be extended for doses missed due to management of toxicities.

If a patient previously dose-reduced completes a treatment cycle without toxicity then escalation of IL-2 dose at next cycle may occur by an increment of 1.5 MIU/dose.

### 2. Antiretroviral Therapy

Antiretroviral drugs will be managed according to good clinical practice. There will be no restrictions on access to antiretroviral therapy during the course of this study. The decision to initiate antiretroviral therapy should be made upon sound clinical evidence that doing so would be in the interest of the patient, their health and well-being.

Optimally, changes to antiretroviral therapy should be made at least 2 weeks following completion of IL-2 therapy and at least 2 weeks prior to commencement of the next cycle. The rationale for change in antiretroviral therapy will be noted clearly in the case report forms.

Patients receiving IL-2 therapy are not required to be on antiretrovirals during the time of IL-2 administrration under this protocol.

### D. CONCOMITANT THERAPY

### 1. Antiretroviral Therapy

Antiretroviral therapy is not a mandatory component of this study. Investigational teams will monitor patient performance at regular intervals to determine the needs of individual patients in terms of antiretroviral therapy

### 2. Antimicrobial Prophylaxis

Patients whose CD4+ cell count falls to below 200 cells/mm^3^ should commence prophylaxis for *Pneumocystis carinii* pneumonia. In patients with a reduction in CD4+ cell count to less than 100 cells/mm^3^ and 50 cells/mm^3^ prophylaxis for *Mycobacterium* *avium* complex and Cytomegalovirus disease respectively, may be initiated. Patients with a history of recurrent Herpes simplex infection who are in receipt of suppressive therapy with acyclovir at commencement of study therapy may continue to receive prophylaxis.

### 3. Treatment of Intercurrent Illnesses

Patients who develop intercurrent illnesses during the trial may receive appropriate systemic therapy followed by appropriate suppressive therapy thereafter. Treatment with IL-2 may be delayed for the management of acute signs and symptoms of intercurrent illnesses as well as toxicities arising from antiretroviral therapy or IL-2. Generally the maximum interval between treatment cycles should not be extended by more than 11 calendar weeks.

### 4. Symptomatic Management During IL-2 Treatment

The use of symptomatic medications during treatment with IL-2 is encouraged. A list of appropriate interventions of this nature is presented in Appendix F.

# VIII. STUDY PROCEDURES

### A. General

Written informed consent must be obtained prior to any study specific tests or evaluations being performed. Additional laboratory tests to those identified in this protocol, necessary to assess patient safety may be performed at the discretion of the investigator. Any other tests should be approved by the appropriate Institutional Review Committee and presented to the potential participant in the informed consent.

Scheduled visits may be adjusted to accommodate holidays. Flexibility of scheduling will generally be at the discretion of the investigator who must determine that patient care and safety will not be compromised. Delay of treatment cycles by more than two weeks from scheduled dates is to be avoided.

### B. Pre-therapy (all patients)

All pre-therapy evaluations must be conducted within 30 days prior to randomization. There will be three pre-study visits required prior to commencement of IL-2 therapy. Patients randomized to receive IL-2 therapy should commence the first treatment cycle within 45 days of pre-therapy visit 1.

#### 1. Visit 1

- provision of written informed consent
- complete medical history and physical examination, weight, blood pressure, respiration rate and temperature (hereafter referred to as **vital signs**)
- chest X-ray and electrocardiogram (unless results are available from studies undertaken within 3 months of assessment date)
- Karnofsky Performance Status (see appendix A)
- laboratory tests to include the following:

i) complete blood count with differential including platelets

ii) serum chemistries to include electrolytes, creatinine, glucose, Ca, Mg, phosphate, AST, ALT, alkaline phosphatase, albumin and bilirubin (hereafter referred to as **serum chemistries**)

iii) flow cytometry panel (appendix G)

iv) serum or urine pregnancy test for women

v) HIV-1 ELISA and confirmatory test if not already available

vi) plasma storage for HIV load testing at central laboratory (appendix H)

vii) whole blood for PBMC processing/storage

#### 2. Visit 2

All results from pre-therapy visit 1 should be available.

- CBC, with differential
- flow cytometry panel
- randomize patient (conact Data Management Centre)
- plasma storage for HIV load testing
- whole blood for PBMC processing/storage

#### 3. Pre-cycle IL-2 (day 1/cycle 1) - Patients randomized to IL-2

This visit must occur within 96 hours of commencement of IL-2, for IL-2 therapy. The following tests must be completed and the results known to be in keeping with study eligibility criteria before the patient is treated with IL-2:

- physical examination, vital signs and Karnofsky Performance Status
- CBC, differential and chemistries
- urinalysis
- urine or serum pregnancy test for women of childbearing potential
- flow cytometry panel
- whole blood for PBMC processing/storage

### C. On study evaluations

Once randomized, patients will be treated with SC IL-2 (IL-2 treated) or no therapy (control). Follow-up and clinical assessments will be different for these patient groups. All on-study assessments are delineated in the following text and tables.

#### 1. IL-2 treated patients cycle 1, 2 and 3.

Patients should have cycle 1 IL-2 administered by a qualified health care professional. Patients should be taught, where possible, how to self administer therapy and should also be educated about the use of prophylactic medications for the management of side effects. The decision to allow the patient to self-administer IL-2 in subsequent cycles should be made by the investigational team.

**Table 3**

**CYCLE 1, 2 and 3 STUDY SCHEMA FOR IL-2 TREATED PATIENTS**

| Test/Assessment/Sample | Pre-cycle IL-2 ^(1)^ | D1 | D2 | D3 | D4 | D5 | D8^(6)*^ | D29^(6)^ |
| --- | --- | --- | --- | --- | --- | --- | --- | --- |
|  |  |  |  |  |  |  |  |  |
| SC IL-2 dosing |  | X | X | X | X | X |  |  |
| Toxicity/ adverse events^(2)^ |  | X | X | X | X | X | X | X |
| Vital signs/ Karnofsky Performance Status | X |  |  |  |  |  |  | X |
| Physical examination^(3)^ | X |  |  |  |  |  | X | X |
| CBC with differential and platelets^(5)^ | X |  |  |  |  | X^(4)^ |  | X |
| Serum chemistries^(5)^ | X |  |  |  |  | X^(4)^ |  | X |
| Pregnancy test-women only ^(5)^ | X |  |  |  |  |  |  |  |
| Flow cytometry | X |  |  |  |  |  |  | X |
| Plasma virology | X |  |  |  |  |  | X | X |
| Whole Blood for PBMC | X |  |  |  |  |  |  |  |

^(1)^ All safety, virological and immunological laboratories must be performed within 96 hours prior to dosing.

^(1)^ The results of all safety evaluations must be known before therapy may commence.

^(2)^ Patients will document adverse experiences on diary cards on an ongoing basis.

^(3)^ A complete physical exam will be performed prior to cycle 1. Thereafter physical exams will be symptom directed or as medically indicated.

^(4)^ Can be obtained on day 4 or 5.

^(5)^ Obtain during pre-therapy visit and immediately prior to dosing for each cycle.

^(6)^ May be±5 days.

**^*^These tests need be performed during administration of the first cycle of IL-2 only.**

Other laboratory testing consistent with patient safety, approved by the EthicsÕ Committee and discussed in the informed consent, may be performed on a site-specific basis.

#### 2. Control patients

In general these patients will require far less intensive monitoring than IL-2 treated patients. Nonetheless, it is important that control patients are reviewed at regular intervals to ensure continuity of follow-up and in an effort to curtail premature withdrawal from study.

Following assignment to this treatment arm, control patients should be reviewed as indicated in Table 4:

**Table 4**

**ANTIRETROVIRAL TREATED PATIENTS**

| Test/assessment/  sample | Week 0^(1)^ | Week 8 ^(1)^ | Week 16 ^(1)^ | Week 24 ^(1)^ |
| --- | --- | --- | --- | --- |
|  |  |  |  |  |
| Vital signs/weight | X | X | X | X |
| History ^(2)^ | X | X | X | X |
| CBC/differential | X | X | X | X |
| Serum chemistries | X | X | X | X |
| Flow cytometry | X | X | X | X |
| Plasma virology | X | X | X | X |
| Whole Blood for PBMC | X | X | X | X |
| Pregnancy test- women only | X |  |  |  |

^(1)^ These visits may take place ± 3 weeks

^(2)^ Update treatment history; verification that no important events occurred

### D. Post-therapy evaluations

Post-therapy evaluations will be conducted on all patients. These evaluations must be completed in the time frame indicated by the following instructions:

- IL-2 treated patients who complete 3 cycles of therapy as described by this protocol should commence post-therapy evaluations no earlier than 8 calendar weeks after commencing the final cycle of IL-2.
- patients who have permanently discontinued IL-2 prematurely as a consequence of toxicity or any other reason, should commence post-therapy evaluations no sooner than 8 weeks after commencement of their most recent treatment cycle.
- control patients should commence post study evaluations no earlier than 24 weeks after entering study.

Post therapy evaluations consist of tests, procedures and samples as shown in Table 5:

**Table 5. Post-therapy evaluations.**

| Test/Assessment/Sample | Post-Study Visit 1 | Post-Study Visit 2 |
| --- | --- | --- |
|  |  |  |
| Physical examination |  | X |
| Vital signs/Karnofsky Performance Status |  | X |
| CBC, with differential and platelet count | X | X ^(1)^ |
| Serum chemistries | X | X ^(1)^ |
| Flow cytometry | X | X ^(1)^ |
| Plasma virology sample | X | X ^(1)^ |
| Whole Blood for PBMC’s | X | X ^(1)^ |

^(1)^ These procedures are to be completed within 2-14 days after commencement of post-therapy visit 1.

### E. Statistical considerations

### 1. Endpoints

The primary endpoint will be the time-weighted mean change in CD4+ cell count from baseline and time-weighted mean change in plasma HIV-RNA from baseline.

Secondary endpoints will include safety data.

### 2. Sample size

Based on an estimate of within patient variability corresponding to a standard deviation of 150 cells/mm^3^ (data on file), and using a two-sided significance level of 5%, a total of 36 patients randomized in the ratio of 2 SC IL-2:1 control participant will give 80% power of detecting an increase in the SC IL-2 group of 150 cells/mm^3^ (175 cells/mm^3^ with 20% dropout).

Based upon an estimate of within patient variability corresponding to a standard deviation of 0.7 log copies/ml (Eron J et al, NEJM 1995; 333:1662-9), and using a two sided significance level of 5%, a total of 36 patients randomised in the ratio of 2 SC IL-2: 1 control participant will give 80% power to detect a 0.7 log copies/ml difference between treatment groups (0.8 log copies/ml with 20% dropout).

### 3. Analysis of data

The final analysis will be conducted once all randomized patients have completed completed the study as described OR one year following initiation of the study. There will be no interim analyses of efficacy data for the purposes of early study termination.

a) Patient characteristics

Patient characteristics at baseline will be summarized by randomized treatment using appropriate summary measures. Patient characteristics to be summarized will include age, sex, baseline CD4+ cell count and virus load. There will be no formal comparisons of baseline characteristics performed.

b) Efficacy parameters

All efficacy data will be analyzed by the intent-to-treat principle. In particular, all doses of SC IL-2 used in the escalation phase will be included in the data from treated patients.

The change from baseline in CD4+ cell count and virus load will be summarized at each sampling time point. Formal analysis will compare the randomized treatments in terms of time-weighted changes from baseline using ANOVA methods, or equivalent non-parametric methods as appropriate.

c) Safety

Safety data will be summarized by treatment received for each time point.

### F. Long term management

Following completion of this protocol all patients should continue in follow-up. An additional protocol will be undertaken to provide continuing treatment to patients on the basis of their original randomization.

# IX. STUDY WITHDRAWAL

### A. Early termination of therapy

Patients will have their study therapy withdrawn if any of the following events occur:

i) withdrawal of consent to participate.

ii) termination of the study.

iii) pregnancy.

iv) a medically significant, grade IV toxicity occurs that is attributable to study therapy.

v) the patient experiences dose-limiting toxicities at the minimal permissible dose for this protocol (1.5 MIU q12h for 5 days every eight weeks).

vi) any event, other than those allowed that delays the commencement of an IL-2 treatment cycle by more than eight weeks.

vii) malignancy requiring systemic therapy.

viii) patient non-compliance.

ix) the principal investigator believes it is in the best interests of the patient.

# X. TRIAL MANAGEMENT

### A. DATA SAFETY MONITORING BOARD

An independent Data Safety Monitoring Board (DSMB) will be established to review the conduct of this study. The DSMB will be asked to address specific questions relevant to the conduct of the trial. The DSMB will make recommendations regarding further progress for the study.

### B. ENDPOINT REVIEW COMMITTEE

An independent Endpoint Review Committee (ERC) will be established to review all diagnostic information provided by participating sites for trial patients who are believed to have had clinical disease. Only those endpoints described as fulfilling all necessary diagnostic criteria by the ERC will be included in the analyses of clinical efficacy. A listing of acceptable definitive and presumptive diagnostic criteria for AIDS-defining illnesses is given in appendix I.

### C. NATIONAL STEERING COMMITTEE

Where appropriate a National Steering Committee will be established. Regular communication within a participant territory will be facilitated by the establishment of a National Steering Committee (NSC). This will include the principal investigator from each site (or their deputy) along with representatives of the IND Sponsors or their agent. This committee will also establish policy for the publication of trial data.

### D. INTERNATIONAL STEERING COMMITTEE

Overall management of the study will be undertaken by the International Steering Committee (ISC). This group will comprise representatives selected from each of the NSC together with representatives from the IND Sponsors. As required the group may also seek the input of *ad hoc* members. This committee will also establish policy for the publication of trial data.

# XI. ADVERSE EVENTS AND MANAGEMENT

### A. ADVERSE EVENT DEFINITION

All adverse events, regardless of severity or relationship to study product, must be recorded in the case report form.An adverse event is any undesired, noxious or pathological change in a patient as indicated by physical signs, symptoms and/or laboratory changes that occurs in association with the use of study drug regardless of the relationship to study drug. This definition includes intercurrent illness or injury, as well as exacerbation of pre-existing conditions, and clinically significant changes in laboratory test results (those that required a change in study therapy). All adverse events should be followed until a final outcome can be achieved, be this resolution or determination that the sequelae are considered chronic. Laboratory abnormalities or changes in weight, Karnofsky Performance Status, or vital signs that are not associated with medical interventions should not be recorded in the adverse event pages. Similarly, conditions/abnormalities arising from infectious origin or from progression of HIV disease (excluding the development of neoplasia) should not be recorded on the adverse pages.

**Please refer to the toxicity grading scale, Appendix D, when assessing the severity of adverse events. Please note, this scale serves as a guide and should be used in association with the patientÕs clinical presentation in any assessment of severity.**

Where a diagnosis is possible it is preferable to report this rather than a series of individual terms each related to the same diagnosis. When reporting a syndrome, indicate the associated signs and symptoms parenthetically following the syndrome rather than as separate events.

Safety will be assessed in all patients enrolled on the study during the treatment period and through the post-treatment evaluation. Any unresolved adverse events or toxicities will be followed until they resolve or until they are determined to be chronic. Any serious adverse events or toxicities that occur during therapy or in 6 months following completion of IL-2 therapy must be reported to the IND Sponsors immediately.

The definition of a serious adverse event is as follows:

A Serious Adverse Event (SAE) means any experience that suggests a significant hazard, contraindication, side effect or precaution. In that regard, medical judgment is required in the evaluation of incoming information. As a rule, a serious adverse event includes any event that is fatal or life-threatening, is permanently disabling, requires or prolongs inpatient hospitalization, is a congenital abnormality in a newborn or requires intervention to prevent permanent impairment or damage. Therefore, the following must be reported:

1. Death

2. Life-threatening illness

3. Hospitalization (initial or prolonged)

4. Disability (results in a significant, persistent, or permanent change, impairment, damage or disruption in the patientÕs body function/structure, physical activities or quality of life)

5. Congenital anomaly in a newborn following treatment during pregnancy

6. Requires intervention to prevent permanent impairment or damage (a condition which requires medical or surgical intervention to preclude permanent impairment or damage to the patient)

7. Occurrence of malignancy

8. Accidental or intentional overdose with sequelae

9. A grade IV toxicity

Any serious adverse event, whether or not considered related to study medication must be reported as soon as possible to the Division of AIDS (DAIDS).

The patient should be followed carefully until the condition has resolved and/or etiology has been identified. Any medication taken to relieve symptoms of the medical problem and the outcome of the event will be recorded.

The Investigator is responsible for informing the IRB as soon as possible, but in no event later than 10 working days after the discovery of death from any cause, or after the discovery of any serious adverse events or problems of clinical significance that may reasonably be regarded as caused or associated with the study product.

### B. DEFINITION OF DOSE-LIMITING ADVERSE EVENT

A medically significant, dose-limiting adverse event will be defined as one which is dose-limiting for a monitored outpatient regimen. All adverse events which are regarded as dose-limiting will be clearly identified in the case report forms. All adverse events will be recorded on case report forms regardless of the assessment of their relation to the drug. **The study monitor must be notified of each dose-limiting adverse event.**

### C. OVERDOSE

In the event the patient receives more IL-2 than the established dosing regimen for a cycle, the study monitor will be notified immediately.

# XII. INSTITUTIONAL REVIEW BOARD APPROVAL/ETHICS COMMITTEE ACCEPTANCE

The principal Investigator agrees to provide the Institutional Review Board (IRB)/Ethics Committee (EC) with all appropriate material including the Informed Consent document. The trial will not be initiated until appropriate IRB/EC approval of the protocol and the Informed Consent document have been obtained in writing by the Investigator and copies have been received by the IND Sponsors. Appropriate reports on the progress of the study by the principal Investigator will be made to the IRB/EC and the IND Sponsors in accordance with applicable government regulations and in agreement with policy established by the IND Sponsors.

# XIII. INFORMED CONSENT

A properly executed written informed consent, in compliance with 21 CFR 50 (see appendix J) as far as is possible within the territory in which the protocol is active, shall be obtained from each patient prior to entering the patient into the trial or prior to performing any unusual or non-routine procedure that involves risk to the patient. If the patient is unable to give consent, he/she may not be entered to the study. A copy of the informed consent document to be used will be submitted by the Investigator to the Institutional Review Board/Ethics Committee (IRB/EC) for review and approval prior to the start of the study. The Investigator shall provide a copy of the signed informed consent to the patient and a copy shall be maintained in the patientÕs record file. Attention is directed to the basic elements that are required to be incorporated into the informed consent under U.S. Federal Regulations for Protection of Human Subjects (21 CFR 50.25[a]). Additional elements of informed consent, if appropriate, must be included in the informed consent document (21 CFR 50.25[b]).

Before initiation of the study, the Investigator must provide the IND Sponsors with a copy of the IRB/EC approved consent form.

# XIV. COMPENSATION, INSURANCE AND INDEMNITY

# XV. PROTOCOL AMENDMENTS

Only the IND Sponsors may modify the protocol. Amendments to the protocol will only be made after consultation and agreement between IND Sponsors and Investigator. The only exception is where the Investigator considers that a patientÕs safety is compromised without immediate action. In these circumstances, immediate approval of the chairman of the IRB/EC must be sought, and the Investigator should inform the IND Sponsors and the full IRB/EC within 5 working days after the emergency occurred. All amendments that impact upon patient risk, the study objectives or require revision of the informed consent document must receive approval from the IRB/EC prior to their implementation.

# XVI. DRUG ACCOUNTABILITY

The Investigator must maintain accurate records recording dates and quantities of product(s) received, to whom dispensed (patient-by-patient accounting) and accounts of any product accidentally or deliberately destroyed. The Investigator must retain all unused or expired product until the study monitor has confirmed accountability data.

# XVII. LABORATORY REQUIREMENTS

Documentation regarding certification, accreditation, or membership of Quality Control Schemes shall be provided to the IND Sponsors for all clinical laboratories utilized for this study. Documentation regarding normal lab ranges must also be provided to the IND Sponsors.

# XVIII. CASE REPORT FORMS

Case Report Forms (CRF) will be provided for each patient. Correction to data on CRFs may be made only by putting a line through the incorrect data and writing the correct values, allowing the original text to remain legible. Each correction should be initialed and dated by the person making the change. If corrections are made after review and signature by the Investigator, (s)he must be made aware of the changes and document this awareness by re-signing and dating the CRF.

The policy is that an appropriate proportion of the study data must be verifiable to the source data, which necessitates access to all original recordings, laboratory reports and patient records. The Investigator must therefore agree to allow access to patient records, and source data must be made available for all study data. The patients must also allow access to their medical records, and they will be informed of this and will be signing their agreement when giving informed consent.

# XIX. STUDY MONITORING

All aspects of the study will be monitored by qualified individuals designated by the IND Sponsors. Monitoring will be conducted according to Good Clinical Practice and standard operating procedures for compliance with applicable government regulations. The Investigator agrees to allow these monitors access to the clinical supplies, dispensing and storage areas and to the clinical files of the study patients and, if requested, agrees to assist the monitors. The frequency of monitoring visits will be at a rate agreed by the Investigator and the IND Sponsors.

In certain circumstances, secondary audit may be conducted by members of the IND SponsorsÕs Quality Assurance department. The Investigator will be informed if this is to take place and advised as to the nature of the audit.

Representatives of the FDA and local regulatory authority may also conduct an audit of the study. If informed of such an audit, the Investigator should notify the IND Sponsors immediately.

# XX. RETENTION OF RECORDS

FDA regulations require that an investigator maintain the records of drug disposition, patient exclusion logs, signed consent forms, case report forms, all correspondence, and supporting documentation for a minimum of 2 years after the date a US marketing application has been approved in the indication investigated in this study or, if no application is to be filed, until 2 years after the investigation is discontinued and the FDA notified.

The Investigator may withdraw from the responsibility to maintain records and transfer custody of the records to another person who will accept responsibility for them. Notice of transfer must be given to the IND Sponsors preferably before, and no later than 10 days after transfer.

# XXI. USE OF INFORMATION AND PUBLICATION

It is understood by the Investigator that the information generated in this study will be used by the IND Sponsors in connection with the development of the product and therefore may be disclosed to government agencies in various countries. In order to allow for the use of information derived from the study, it is understood that the investigator is obliged to provide the IND Sponsors with complete test results, all study data, and access to all study records.

The Investigators will be free to publish the results from this study; however, the publication must be in a form which does not reveal technical information provided by the IND Sponsors which is considered confidential or proprietary. A copy of the presentation or publication (including abstracts) must be submitted to the IND Sponsors for pre-approval at least 30 days before its submission to any meeting or journal. The publication must also be reviewed by the National Steering Committee and the International Steering Committee at least 30 days before submission to any journal or meeting secretariat.

# XXII. REFERENCES

Deresinski SC, Israelski DI, Frascino RJ, *et al*. Randomized multicentre comparison of treatment of HIV infected patients. Activated CD8+ cells plus rIL-2 plus antiretroviral (AR) therapy vs rIL-2 plus AR vs AR alone. *2^nd^ National Conf on Human Retroviruses*, Washington DC. February 1995. Abstract 206.

Kovacs JA, Baseler M, Dewar RJ, *et al.* Increases in CD4 T lymphocytes with intermittent courses of interleukin-2 in patients with human immunodeficiency virus infection. A preliminary study. *N Engl J Med*  1995; 332:567-575.

Kovacs JA, Vogel S, Albert J, *et al.* Randomized trial of intermittent interleukin-2 therapy in HIV-infected patients with CD4 counts>200 cells/mm3.*N Eng J Med*  1996: 335:1350-6

Lane HC, *et al.* Use of interleukin-2 in patients with acquired immunodeficiency syndrome. *J Biol Resp Modifiers*  1984; 3:512-516.

Rook AH, Masur H, Lane HC, *et al* . Interleukin-2 specifically enhances the depressed natural killer and cytomegalovirus-specific cytotoxic activities of lymphocytes from patients with the acquired immunodeficiency syndrome. *J Clin Invest*  1985; 72: 398-403.

Smith KA. Interleukin-2: Inception, impact and implications. *Science*  1988; 240: 1169-1176.

Davey RT, Chaitt DG, Piscitelli SC, *et al.* Subcutaneous Administration of Interleukin-2 in Human Immunodeficiency Virus Type-1Infected Persons. *J Infect Dis* 1997; 175:781-9.

**APPENDIX A: KARNOFSKY PERFORMANCE STATUS PERFORMANCE STATUS CRITERIA**

100 Normal; no complaints.

90 Able to carry on normal activities; minor signs or symptoms of disease.

80 Normal activity with effort.

70 Cares for self. Unable to carry on normal activity or to do active work.

60 Ambulatory. Requires some assistance in activities of daily living and self care.

50 Requires considerable assistance and frequent medical care.

40 Disabled; requires special care and assistance.

30 Severely disabled; hospitalization indicated though death not imminent.

20 Very sick; hospitalization and active supportive treatment.

10 Moribund.

0 Dead.

**APPENDIX B: CDC 1993. CASE DIAGNOSES - AIDS**

**1993 REVISED CLASSIFICATION SYSTEM FOR HIV INFECTION AND EXPANDED SURVEILLANCE CASE DEFINITION FOR AIDS AMONG ADOLESCENTS AND ADULTS**

The revised CDC classification system for HIV-infected adolescents and adults categorizes persons on the basis of clinical conditions associated with HIV infection and CD4+T lymphocyte counts. The system is based on three ranges of CD4+T-lymphocyte counts and three clinical categories and is represented by a matrix of nine mutually exclusive categories (Table 1).

**Criteria for HIV infection for persons ages >13 years:**

a) Repeatedly reactive screening tests for HIV antibody (e.g. enzyme immunoassay) with specific antibody identified by the use of supplemental tests (e.g.Western blot, immunofluorescence assay) or;

b) direct identification of virus in host tissues by virus isolation or;

c) HIV antigen detection; or

d) a positive result on any other highly specific licensed test for HIV.

**Table 1. 1993 revised classification system for HIV infection and expanded AIDS surveillance case definition for adolescents and adults***

| **CD4+ T-cell categories** | **Clinical categories** | | |
| --- | --- | --- | --- |
|  | (A)  Asymptomatic, acute (primary) HIV or PGL | (B)  Symptomatic, not (A) or (B) conditions^¤^ | (C)  AIDS-indicator conditions^¦^ |
| (1)>500/µL | A1 | B1 | C1 |
| (2)200-499/µL | A2 | B2 | C2 |
| (3)<200/µL AIDS indicator T-cell count | A3 | B3 | C3 |

*The shaded cells illustrate the expanded AIDS surveillance case definition.

  PGL:persistent generalized lymphadenopathy. Clinical Category A includes acute (primary) HIV infection.

¤See text for discussion

¦ See list of conditions included in the 1993 AIDS surveillance case definition

**CD4+ T-lymphocyte Categories**

The three CD4+ T-lymphocyte categories are defined as follows:

**Category 1:** >500 cells/µL

**Category 2:** 200-499 cells/µL

**Category 3:** <200 cells/µL

HIV-infected persons should be classified according to the lowest accurate, but not necessarily the most recent CD4+T-lymphocyte count.

**Equivalences for CD4+ T-lymphocyte count and percentage of total lymphocytes.**

The CD4+ percentage is less subject to variation on repeated measurements when compared with the absolute CD4+ count. The 1993 classification emphasizes the use of absolute CD4+ T-lymphocyte counts but allows for the use of CD4+ percentages, based on large multicenter studies results.

The best concordance between CD4+ percentage and an absolute CD4+ T-lymphocyte count was found when the percentage was <14 , corresponding to <200/µL in 90.2% of the cases. See table below for other suggested equivalences.

**Table 2. Equivalences for absolute numbers of CD4+ T-lymphocytes and CD4+ percentage (the percentage of lymphocytes that are CD4+ T-cells)**

| CD4+ T-cell category | CD4+ T-cell/µL | CD4+ percentage |
| --- | --- | --- |
| (1) | ³500 | ³29 |
| (2) | 200-499 | 14-28 |
| (3) | <200 | <14 |

**Clinical Categories**

The clinical categories of HIV infection are defined as follows:

**Category A**

It includes one or more of the following conditions in an HIV infected person (³13 years old). None of the conditions listed in Categories B and C must have occurred.

- Asymptomatic HIV infection
- Persistent generalized lymphadenopathy
- Acute (primary) HIV infection with accompanying illness or history of acute HIV infection

**Category B**

It consists of symptomatic conditions that are not listed in clinical Category C and that meet at least one of the following criteria:

a) They are attributed to HIV infection or are indicative of a defect in cell-mediated immunity

b) conditions whose clinical course or management is thought to be complicated by HIV infection. Examples of category B conditions are but they are not limited to:

- Bacillary angiomatosis
- Oropharyngeal candidiasis (thrush)
- Vulvovaginal candidiasis that is persistent, frequent or poorly responsive to therapy
- Cervical dysplasia (moderate or severe)/cervical carcinoma in situ
- Constitutional symptoms, such as fever (38.5¼C) or diarrhea lasting > 1 month
- Hairy leukoplakia, oral
- Herpes zoster (shingles), involving at least two distinct episodes or more than one dermatome.
- Idiopathic thrombocytopenic purpura
- Listeriosis
- Pelvic inflammatory disease, particularly if complicated by tubo-ovarian abscess
- Peripheral neuropathy

Category B conditions take precedence over those in Category A.

If someone is now asymptomatic but has had any of the Category B conditions, that person should be classified in Category B, unless he has developed a Category C condition, in which case that person will belong to Category C.

**Category C**

Conditions that are AIDS case definition as of January 1^st^ 1993:

- Candidiasis of bronchi, trachea or lungs
- Candidiasis, esophageal
- Cervical cancer, invasive
- Coccidioidomycosis, disseminated or extrapulmonary
- Cryptococcosis, extrapulmonary
- Cryptosporidiosis, chronic intestinal
- Cytomegalovirus disease (other than liver, spleen or nodes)
- Cytomegalovirus retinitis (with loss of vision
- Encephalopathy, HIV-related
- Herpes simplex: chronic ulcer (>1 month duration); or bronchitis, pneumonitis, or esophagitis
- Histoplasmosis, disseminated or extrapulmonary
- Isosporiasis, chronic, intestinal
- KaposiÕs sarcoma
- Lymphoma, BurkittÕs (or equivalent term)
- Lymphoma, immunoblastic (or equivalent term)
- Lymphoma, primary, of brain
- Mycobacterium avium complex or M.kansasii, disseminated or extrapulmonary
- Mycobacterium tuberculosis, any site (pulmonary or extrapulmonary)
- Mycobacterium, other species or unidentified species, disseminated or extrapulmonary
- Pneumocystis carinii pneumonia
- Pneumonia, recurrent (two or more episodes over a 1-year period)
- Progressive multifocal leukoencephalopathy
- Salmonella septicemia, recurrent (two or more episodes over a 1-year period)
- Toxoplasmosis of brain
- Wasting syndrome due to HIV
- American Trypanosomiasis (Chagas Disease) of the Central Nervous System

**Definitive diagnostic methods for diseases indicative of AIDS**

**Diseases Diagnostic methods**

Cryptosporidiosis Microscopy (histology or cytology)

Isosporiasis

KaposiÕs sarcoma

Lymphoma

Pneumocystis carinii pneumonia

Progressive multifocal leukoencephalopathy

Toxoplasmosis

Cervical cancer

Chagas disease of the CNS

Candidiasis Gross inspection of tissues (by endoscopy or autopsy) or by microscopy (histology or cytology) on the tissues affected, not from a culture.

Coccidioidomycosis Microscopy (histology or cytology),

or Cryptococcosis culture, antigen detection in the Cytomegalovirus tissues affected or in a fluid from those tissues.

Herpes simplex virus

Histoplasmosis

Tuberculosis Culture.

Other mycobacteriosis

Salmonellosis

HIV encephalopathy Cognitive or motor dysfunction progressing (dementia)over weeks to months and interfering with daily living or occupation, in the absence of a concurrent illness or condition other than HIV infection. Those other causes must be excluded by cerebrospinal fluid examination, brain imaging (computed tomography magnetic resonance) or autopsy.

HIV wasting syndrome Involuntary weight loss of >10% of baseline body weight plus either chronic diarrhea (at least two loose stools per day for >30 days, or chronic weakness and documented fever (for >3days, intermittent or constant) in the absence of a concurrent illness or condition other than HIV infection that could cause the syndrome (e.g. cancer, tuberculosis, cryptosporidiosis, or other specific enteritis).

Pneumonia, recurrent More than one episode in a 1-year period, acute (new x-ray evidence) diagnosed by both: a) culture (or other organism specific method) obtained from a clinically reliable specimen of a pathogen that typically produces pneumonia (other than *Pneumocystis carinii* or *Mycobacterium tuberculosis* and b) radiologic evidence of pneumonia; cases that do not have laboratory confirmation of a causative organism for one of the episodes of pneumonia will be considered to be presumptively diagnosed.

**Suggested guidelines for presumptive diagnosis of diseases indicative of AIDS.**

**Diseases Presumptive criteria**

Candidiasis of a. Recent onset of retrosternal esophagus pain on swallowing;

##### AND

b. Oral candidiasis diagnosed by the gross appearance of white patches or plaques on an erythematous base or by the microscopic finding of fungal mycelial filaments in a scraped sample from the oral mucosa.

Cytomegalovirus Characteristic findings on serial ophtalmoscopic examinations (e.g., discrete patches of retinal whitening with borders, spreading in a centrifugal manner along the paths of blood vessels, progressing over several months, and frequently associated with retinal vasculitis, hemorrhage, and necrosis). Resolution of active disease leaves retinal scarring atrophy with retinal pigment epithelial mottling.

Mycobacteriosis Microscopy of a specimen from a site other than the lungs, skin, or cervical or hilar lymph nodes that shows acid fast bacilli not identified by culture.

KaposiÕs sarcoma Erythematous or violaceous plaque-like lesion on skin or mucous membrane.

*Pneumocystis carinii* a. Dyspnea on exertion or nonproductive cough pneumonia of recent onset (within the three months)

AND

b. Evidence of diffuse bilateral interstitial infiltrates by either chest x-ray or gallium scan.

###### AND

c. A pO_2_ of <70 mmHg from arterial blood gas analysis or a low respiratory diffusing capacity (<80% of predicted values) or an increase in the alveolar-arterial oxygen tension gradient;

AND

d. No evidence of a bacterial pneumonia.

Pneumonia, recurrent More than one episode in a 1-year period, acute (new symptoms, signs or x-ray findings not present earlier) pneumonia diagnosed on clinical or radiologic grounds by the physician.

Toxoplasmosis of brain a. Recent onset of a focal neurologic abnormality consistent with intracranial disease or a reduced level of consciousness;

###### AND

b.Brain imaging evidence (computed tomography or nuclear magnetic resonance) of a lesion having mass effect or is enhanced by injection of contrast medium;

###### AND

c.Serum antibody to toxoplasmosis or successful response to therapy for toxoplasmosis.

Tuberculosis, pulmonary When bacteriologic confirmation is not available, other reports may be considered to be verified cases of pulmonary tuberculosis if the criteria for clinical case definition of the CDC are used. These include:

a. A positive tuberculin test b. Other signs and symptoms compatible with tuberculosis, such as an abnormal, unstable (worsening or improving) chest x-ray, clinical evidence of current disease c. Treatment with two or more antituberculosis medications, and d. Completed diagnostic evaluation. ( MMWR 1990; 39 (No.RR-13): 39- 40).

### APPENDIX C. INSTRUCTIONS FOR RECONSTITUTION OF IL-2 FOR ADMINISTRATION

**RECONSTITUTION OF IL-2**

The following information applies to dosage preparation for the Interleukin-2 (IL-2) formulation dispensed for this protocol only. The vials each contain a total of **21.6** million international units (MIU) of interleukin-2, and are labelled as Ò**SC RIL-2 Injection**Ó. To prepare single injectable doses at each of these dose levels, reconstitute with **sterile water** and withdraw the respective volumes as follows:

| Dose  (MIU) | Volume  sterile H_2_O (ml) | Final  concentration | Volume  per dose (ml) |
| --- | --- | --- | --- |
|  |  |  |  |
| **1.5** | **2.5** | **8.64MIU/ml** | **0.17** |
|  |  |  |  |
| **4.5** | **2.5** | **8.64MIU/ml** | **0.52** |
|  |  |  |  |
| **7.5** | **2.5** | **8.64MIU/ml** | **0.87** |
|  |  |  |  |
|  |  |  |  |

**APPENDIX D. CPCRA TOXICITY TABLE FOR GRADING ADVERSE EVENTS**

The following table will be used for reporting adverse events as defined in protocol section XI (A). All adverse events, regardless of severity or relationship to study drug, must be reported in the case report form**.**

***All Grade 4 toxicities require that study drug be held until the toxicity decreases in severity to < Grade 2. Those toxicities which require that study drug be held before Grade 4 occurs are shaded (see Appendix E for IL-2 dose reduction guidelines for these shaded toxicities).**

***All Grade 4 toxicities must be reported as soon as possible to DAIDS as outlined in Section XI(A) of the protocol.**

Estimating severity grade

For abnormalities NOT found elsewhere on the Toxicity Table use the scale below to estimate grade of toxicity:

GRADE 1 Transient or mild discomfort; no limitation in activity; no medical intervention/therapy required.

GRADE 2 Mild to moderate limitation in activity - some assistance may be needed; no or minimal medical intervention/therapy required.

GRADE 3 Marked limitation in activity, some assistance usually required; medical intervention/therapy required, hospitalization possible.

GRADE 4 Extreme limitation in activity, significant assistance required; significant medical intervention/therapy required, hospitalization or hospice care

| **HEMATOLOGY** | **GRADE 1** | **GRADE 2** | **GRADE 3** | **GRADE 4** |
| --- | --- | --- | --- | --- |
| Hemoglobin | 8.0 - 9.4 g/dL OR  80 - 94 g/L OR  4.93 - 5.83 mmol/L | 7.0 - 7.9 g/dL OR  70-79 g/L OR  4.31 - 4.92 mmol/L | 6.5 - 6.9 g/dL OR  65 - 69 g/L OR  4.03 - 4.30 mmol/L | <6.5 g/dL OR  <65 g/L OR  <4.03 mmol/L |
| Absolute Neutrophil Count | 1000 - 1500/mm^3^ OR  1.0 - 1.5/G/L* | 750 - 999/mm^3^ OR  0.75 -0.99/G/L* | 500 - 749/mm^3^ OR  0.5 - 0.749/G/L* | <500/mm^3^ OR  <0.5/G/L* |
| Platelets | 75,000 - 99,000/mm^3^ OR  75 - 99/G/L* | 50,000 - 74,999/mm^3^ OR  50 - 74.9/G/L* | 20,000 - 49,999/mm^3^ OR  20 - 49.9/G/L* | <20,000/mm^3^ OR  <20/G/L* |
| Prothrombin Time (PT) | >1.0 - 1.25 X ULN | >1.25 - 1.5 X ULN | >1.5 - 3.0 X ULN | >3.0 X ULN |
| PTT | >1.0 - 1.66 X ULN | >1.66 - 2.33 X ULN | >2.33 - 3.0 X ULN | >3.0 X ULN |
| Methemoglobin | 5.0 - 10.0% | 10.1 - 15.0% | 15.1 - 20.0% | >20% |
| *G = X 10^9^ |  |  |  |  |

| **CHEMISTRIES** | **GRADE 1** | **GRADE 2** | **GRADE 3** | **GRADE 4** |
| --- | --- | --- | --- | --- |
| SODIUM |  |  |  |  |
| Hyponatremia | 130 - 135 meq/L **OR**  130 - 135 mmol/L | 123 - 129 meq/L **OR**  123 - 129 mmol/L | 116 - 122 meq/L **OR**  116 - 122 mmol/L | <116 meq/L **OR**  <116 mmol/L |
| Hypernatremia | 146 - 150 meq/L **OR**  146 - 150 mmol/L | 151 - 157 meq/L **OR**  151 - 157 mmol/L | 158 - 165 meq/L **OR**  158 - 165 mmol/L | >165 meq/L **OR**  >165 mmol/L |
| POTASSIUM |  |  |  |  |
| Hyperkalemia | 5.6 - 6.0 meq/L **OR**  5.6 - 6.0 mmol/L | 6.1 - 6.5 meq/L **OR**  6.1 - 6.5 mmol/L | 6.6 - 7.0 meq/L **OR**  6.6 - 7.0 mmol/L | >7.0 meq/L **OR**  >7.0 mmol/L |
| Hypokalemia | 3.0 - 3.4 meq/L **OR**  3.0 - 3.4 mmol/L | 2.5 - 2.9 meq/L **OR**  2.5 - 2.9 mmol/L | 2.0 - 2.4 meq/L **OR**  2.0 - 2.4 mmol/L | <2.0 meq/L **OR**  <2.0 mmol/L |
| PHOSPHATE |  |  |  |  |
| Hypophosphatemia | 2.0 - 2.4 mg/dL **OR**  0.63 - 0.77 mmol/L | 1.5 - 1.9 mg/dL **OR**  0.46 - 0.62 mmol/L | 1.0 - 1.4 mg/dL **OR**  0.32 - 0.45 mmol/L | <1.0 mg/dL **OR**  <0.32 mmol/L |
| CALCIUM - (corrected for albumin) |  |  |  |  |
| Hypocalcemia | 7.8 - 8.4 mg/dL **OR**  1.95 - 2.10 mmol/L | 7.0 - 7.7 mg/dL **OR**  1.74 - 1.94 mmol/L | 6.1 - 6.9 mg/dL **OR**  1.53 - 1.73 mmol/L | <6.1 mg/dL **OR**  <1.53 mmol/L |
| Hypercalcemia | 10.6 - 11.5 mg/dL **OR**  2.65 - 2.87 mmol/L | 11.6 - 12.5 mg/dL **OR**  2.88 - 3.14 mmol/L | 12.6 - 13.5 mg/dL **OR**  3.15 - 3.37 mmol/L | >13.5 mg/dL **OR**  >3.37 mmol/L |
| MAGNESIUM |  |  |  |  |
| Hypomagnesemia | 1.2 - 1.4 meq/L **OR**  0.60 - 0.70 mmol/L | 0.9 - 1.1 meq/L **OR**  0.45 - 0.59 mmol/L | 0.6 - 0.8 meq/L **OR**  0.30 - 0.44 mmol/L | <0.6 meq/L **OR**  <0.30 mmol/L |
| BILIRUBIN |  |  |  |  |
| Hyperbilirubinemia | >1.0 - 1.5 X ULN | >1.5 - 2.5 X ULN | >2.5 - 5 X ULN | >5 X ULN |
| GLUCOSE |  |  |  |  |
| Hypoglycemia | 55 - 64 mg/dL **OR**  3.01 - 3.55 mmol/L | 40 - 54 mg/dL **OR**  2.19 - 3.00 mmol/L | 30 - 39 mg/dL **OR**  1.67 - 2.18 mmol/L | <30 mg/dL **OR**  <1.67 mmol/L |
| Hyperglycemia (nonfasting and no prior diabetes) | 116 - 160 mg/dL **OR**  6.44 - 8.90 mmol/L | 161 - 250 mg/dL **OR**  8.91 - 13.88 mmol/L | 251 - 500 mg/dL **OR**  13.89 - 27.76 mmol/L | >500 mg/dL **OR**  >27.76 mmol/L |
| TRIGLYCERIDES | --------------------- | 400 - 750 mg/dL **OR**  4.52 - 8.47 mmol/L | 751 - 1200 mg/dL **OR**  8.48 - 13.55 mmol/L | >1200 mg/dL **OR**  >13.55 mmol/L |
| CREATININE | >1.0 - 1.5 X ULN | >1.5 - 3.0 X ULN | >3.0 - 6.0 X ULN | >6.0 X ULN |
| URIC ACID |  |  |  |  |
| Hyperuricemia | 7.5 - 10.0 mg/dL **OR**  446 - 595 umol/l | 10.1 - 12.0 mg/dL **OR**  596 - 716 umol/l | 12.1 - 15.0 mg/dL **OR**  717 - 892 umol/l | >15.0 mg/dL **OR**  >892 umol/l |
| **CHEMISTRIES** (con't.) | **GRADE 1** | **GRADE 2** | **GRADE 3** | **GRADE 4** |
| LIVER TRANSAMINASE (LFTs) |  |  |  |  |
| AST (SGOT) | 1.25 - 2.5 X ULN | >2.5 - 5.0 X ULN | >5.0 - 10.0 X ULN | >10.0 X ULN |
| ALT (SGPT) | 1.25 - 2.5 X ULN | >2.5 - 5.0 X ULN | >5.0 - 10.0 X ULN | >10.0 X ULN |
| GGT | 1.25 - 2.5 X ULN | >2.5 - 5.0 X ULN | >5.0 - 10.0 X ULN | >10.0 X ULN |
| Alk Phos | 1.25 - 2.5 X ULN | >2.5 - 5.0 X ULN | >5.0 - 10.0 X ULN | >10.0 X ULN |
| PANCREATIC ENZYMES |  |  |  |  |
| Amylase | >1.0 - 1.5 X ULN | >1.5 - 2.0 X ULN | >2.0 - 5.0 X ULN | >5.0 X ULN |
| Pancreatic amylase | >1.0 - 1.5 X ULN | >1.5 - 2.0 X ULN | >2.0 - 5.0 X ULN | >5.0 X ULN |
| Lipase | >1.0 - 1.5 X ULN | >1.5 - 2.0 X ULN | >2.0 - 5.0 X ULN | >5.0 X ULN |

| **CARDIO-VASCULAR** | **GRADE 1** | **GRADE 2** | **GRADE 3** | **GRADE 4** |
| --- | --- | --- | --- | --- |
| CARDIAC ARRHYTHMIA | ------------------ | Asymptomatic; transient dysrhythmia, no Rx req | Recurrent/persistent dysrhythmia; symptomatic Rx req | Unstable dysrhythmia, hospitalization and Rx req |
| HYPERTENSION | Transient, increase >20 mmHg; no Rx | Recurrent; chronic increase >20 mmHg, Rx req | Acute Rx req; outpatient hospitalization possible | Hospitalization req |
| HYPOTENSION | Transient orthostatic hypotension, no Rx | Symptoms correctable with oral fluid Rx | IV fluid req, no hospitalization req | Hospitalization req |
| PERICARDITIS | Minimal effusion | Mild/mod asymptomatic effusion, no Rx | Symptomatic effusion, pain, EKG changes | Tamponade **OR** pericardiocentesis **OR** surgery req |
| HEMORRHAGE, BLOOD LOSS | --------------------- | Mildly symptomatic, no Rx required | Gross blood loss **OR** 1-2 units transfused | Massive blood loss **OR** >2 units transfused |

| **GASTROINTESTINAL** | **GRADE 1** | **GRADE 2** | **GRADE 3** | **GRADE 4** |
| --- | --- | --- | --- | --- |
| NAUSEA | Mild **OR** transient; reasonable intake maintained | Mod discomfort **OR** intake decreased for <3 days | Severe discomfort **OR** minimal intake for >3 days | Hospitalization req |
| VOMITING | Mild **OR** transient; 2-3 episodes per day **OR** mild vomiting lasting <1 week | Mod **OR** persistent; 4-5 episodes per day **OR** vomiting lasting >1 week | Severe vomiting of all food/fluids in 24 hrs **OR** orthostatic hypotension **OR** IV Rx req | Hypotensive shock **OR** hospitalization req for IV Rx req |
| DIARRHEA | Mild **OR** transient; 3-4 loose stools per day **OR** mild diarrhea lasting <1 week | Mod **OR** persistent; 5-7 loose stools per day **OR** diarrhea lasting >1 week | Bloody diarrhea **OR** orthostatic hypotension **OR** >7 loose stools/day **OR** IV Rx req | Hypotensive shock **OR** hospitalization req |
| **GASTROINTESTINAL** (con't.) | **GRADE 1** | **GRADE 2** | **GRADE 3** | **GRADE 4** |
| ORAL DISCOMFORT/  DSYPHAGIA | Mild discomfort, no difficulty swallowing | Difficulty swallowing but able to eat and drink | Unable to swallow solids | Unable to drink fluids; IV fluids req |
| CONSTIPATION | Mild | Moderate | Severe | Distention with vomiting |

| **NEUROLOGIC** | **GRADE 1** | **GRADE 2** | **GRADE 3** | **GRADE 4** |
| --- | --- | --- | --- | --- |
| NEURO-CEREBELLAR | Slight incoordination **OR** dysdiadochokinesia | Intention tremor **OR** dysmetria **OR** slurred speech **OR** nystagmus | Locomotor ataxia | Incapacitated |
| NEURO-PSYCH/MOOD | --------------------- | --------------------- | Severe mood changes requiring medical intervention | Acute psychosis req hospitalization |
| PERIPHERAL NEUROPATHY | *Peripheral neuropathy is a syndrome characterized by signs or symptoms of one or several specific neurologic manifestations: neuromuscular paresis, paresthesia, neuromotor, and neurosensory abnormalities.* | *Peripheral neuropathy should be graded as the maximum grade, according to the DAIDS toxicity table, of any of these manifestations which, in your clinical judgment, are due to peripheral neuropathy rather than another (i.e., central) cause.* |  |  |
| NEUROMUSC. PARESIS | Subjective weakness; no objective symptoms/signs | Mild objective signs, symptoms, no decrease in function | Objective weakness; function limited | Paralysis |
| NEURO-MOTOR | Decrease in reflexes **OR** patients with chronic stable abnormality of reflex or use of muscle | Absence of a previously present reflex | Absence of 2-3 previously present reflexes | Absence of >3 previously present reflexes |
| NEURO-SENSORY | Decrease in sensation (pinprick, vibratory or hot/cold) **OR** patients with chronic **STABLE** abnormality of sensation | Absence of a previously present sensory finding (one dermatome) | Absence of 2-3 previously present sensory dermatomes | Absence of >3 previously present sensory dermatomes |

| **RESPIRATORY** | **GRADE 1** | **GRADE 2** | **GRADE 3** | **GRADE 4** |
| --- | --- | --- | --- | --- |
| BRONCHOSPASM, Acute | Transient; no Rx; FEV1 <80% - 70% (or peak flow) | Rx req; normalizes with bronchodilator; FEV1 50% - <70% (or peak flow) | No normalization with bronchodilator; FEV1 25% - <50% (or peak flow), retractions | Cyanosis; FEV1 <25% (or peak flow) **OR** intubated |
| DYSPNEA | Dysnea on exertion | Dyspnea with normal activity | Dyspnea at rest | Dyspnea requiring O_2_ therapy |

| **URINALYSIS** | **GRADE 1** | **GRADE 2** | **GRADE 3** | **GRADE 4** |
| --- | --- | --- | --- | --- |
| PROTEINURIA |  |  |  |  |
| Spot urine | 1+ | 2 - 3+ | 4+ | Nephrotic syndrome |
| 24 hour urine | 200 mg-1 g loss/day **OR** <0.3%  **OR** <3 g/l | 1 - 2 g loss/day  **OR** 0.3 - 1.0%  **OR** 3 - 10 g/l | 2 - 3.5 g loss/day **OR** >1.0%  **OR** >10 g/l | Nephrotic syndrome **OR** >3.5 g loss/day |
| GROSS HEMATURIA | Microscopic only | Gross, no clots | Gross plus clots | Obstructive **OR** transfusion req |

| **MISCELLANEOUS** | **GRADE 1** | **GRADE 2** | **GRADE 3** | **GRADE 4** |
| --- | --- | --- | --- | --- |
| FEVER (oral, >12 hours) | 37.7 - 38.5^0^C **OR** 100.0 - 101.5^0^F | 38.6 - 39.5^0^C **OR** 101.6 - 102.9^0^F | 39.6 - 40.5^0^C**OR** 103 - 105^0^F | >40.5^0^C **OR**  >105^0^F |
| HEADACHE | Mild; no Rx req | Moderate **OR** non-narcotic analgesia Rx | Severe **OR** responds to initial narcotic Rx | Intractable **OR** requiring repeated narcotic Rx |
| ALLERGIC REACTION | Pruritus without rash | Localized urticaria | Generalized urticaria angioedema | Anaphylaxis |
| CUTANEOUS/RASH/  DERMATITIS | Erythema, pruritus | Diffuse maculopapular rash **OR** dry desquamation | Vesiculation **OR** moist desquamation **OR** ulceration | **ANY ONE**: mucous membrane involvement, suspected Stevens-Johnson (TEN), erythema multiforme, necrosis req surgery, exfoliative dermatitis |
| LOCAL REACTION (2% parenteral Rx - not vaccination or skin test) | Erythema | Induration <10 mm **OR** inflammation **OR** phlebitis | Induration >10 mm **OR** ulceration | Necrosis of skin |
| FATIGUE | Normal activity reduced <25% | Normal activity reduced 25-50% | Normal activity reduced >50%; cannot work | Unable to care for self |

**APPENDIX E. GUIDELINES FOR IL-2 DOSE REDUCTION**

The toxicities on the following table require IL-2 dose modification. A cycle in which such a toxicity occurs will be held until the toxicity is considered, in the opinion of the principal investigator, to no longer be dose-limiting.

The guidelines below are minimum recommendations and the decision to stop, dose-modify and/or restart study drug should be based on the clinical judgement of the principle investigator in discussion, as appropriate, with other investigators and/or the IND sponsors.

*** All Grade 4 toxicities as defined in Appendix D require that study drug be held until the toxicity decreases in severity to < Grade 2. Guidelines for the management of toxicities shaded in Appendix D (those toxicities which may require that study drug be held before Grade 4 severity occurs) are described below.**

| **HEMATOLOGY** | **HOLD IL-2 FOR:** | **CONSIDER RESTARTING**  **IL-2 FOR:** |
| --- | --- | --- |
| Hemoglobin | < 7.9 g/dL OR  < 79 g/L OR  < 4.92 mmol/L | > 9.4 g/dL OR  > 94 g/L OR  > 5.83 mmol/L |
| Platelets | < 50,000/mm^3^ OR  < 50 /G/L* | > 50,000/mm^3^ OR  > 50 /G/L* |
| Prothrombin Time (PT) | >1.5 X ULN | < 1.25 X ULN |
| PTT | >2.33 X ULN | <1.66 X ULN |
| *G = X 10^9^ |  |  |

| **CHEMISTRIES** | **HOLD IL-2 FOR:** | **CONSIDER RESTARTING**  **IL-2 FOR:** |
| --- | --- | --- |
| SODIUM |  |  |
| Hyponatremia | < 129 meq/L **OR**  < 129 mmol/L | > 130 meq/L **OR**  > 130 mmol/L |
| POTASSIUM |  |  |
| Hypokalemia | <2.5 meq/L **OR**  <2.5 mmol/L | > 3.4 meq/L **OR**  > 3.4 mmol/L |
| CALCIUM - (corrected for albumin) |  |  |
| Hypocalcemia | < 7.0 mg/dL **OR**  < 1.74 mmol/L | > 7.0 mg/dL **OR**  >1.74 mmol/L |
| MAGNESIUM |  |  |
| Hypomagnesemia | <0.60 meq/L **OR**  <0.30 mmol/L | >0.9 meq/L **OR**  >0.45 mmol/L |
| **CHEMISTRIES** (con't.) | **HOLD IL-2 FOR:** | **CONSIDER RESTARTING**  **IL-2 FOR:** |
| GLUCOSE |  |  |
| Hypoglycemia | <40 mg/dL **OR**  <2.19 mmol/L | >64 mg/dL **OR**  >3.55 mmol/L |
| Hyperglycemia (nonfasting and no prior diabetes) | >300 **OR**  > 16.0 mmol/L | <200 mg/dL **OR**  < 11.1 mmol/L |
| CREATININE | >1.5 X ULN | <1.1 X ULN |

| **CARDIOVASCULAR** | **HOLD IL-2 FOR:** | **CONSIDER RESTARTING**  **IL-2 FOR:** |
| --- | --- | --- |
| CARDIAC ARRHYTHMIA | Asymptomatic; transient dysrhythmia, no Rx req | Resolution of dysrythmia |
| HYPERTENSION | Recurrent; chronic increase >20 mmHg, Rx req |  |
| HYPOTENSION | IV fluid required | Return to patientÕs baseline blood pressure |
| PERICARDITIS | Mild/mod asymptomatic effusion | Minimal effusion; no rub. |
| HEMORRHAGE, BLOOD LOSS | Gross blood loss or 1-2 units transfused | No gross blood loss and no transfusion requirements. |

| **GASTROINTESTINAL** | **HOLD IL-2 FOR:** | **CONSIDER RESTARTING**  **IL-2 FOR:** |
| --- | --- | --- |
| NAUSEA | Severe discomfort **OR** minimal intake for >3 days | Mild **OR** transient; reasonable intake maintained |
| VOMITING | Severe vomiting of all food/fluids in 24 hrs **OR** orthostatic hypotension **OR** IV Rx req | Mild **OR** transient; 2-3 episodes per day |
| DIARRHEA | Bloody diarrhea **OR** orthostatic hypotension **OR** >7 loose stools/day **OR** IV Rx req | Mild **OR** transient; 3-4 loose stools per day |
| ORAL DISCOMFORT/  DSYPHAGIA | Unable to swallow solids | Difficulty swallowing but able to eat and drink |

| **NEUROLOGIC** | **HOLD IL-2 FOR:** | **CONSIDER RESTARTING**  **IL-2 FOR:** |
| --- | --- | --- |
| NEURO-CEREBELLAR | Slight incoordination **OR** dysdiadochokinesia | Resolution of symptoms |
| NEURO-PSYCH/MOD | Severe mood changes requiring medical intervention | Resolution of symptoms |
| NEUROMUSCULAR PARESIS | objective weakness, function limited | No objective signs |
| NEURO-MOTOR | Absence of a previously present reflex | Decrease in reflexes **OR** patients with chronic stable abnormality of reflex or use of muscle |

| **RESPIRATORY** | **HOLD IL-2 FOR:** | **CONSIDER RESTARTING**  **IL-2 FOR:** |
| --- | --- | --- |
| BRONCHOSPASM, Acute | Rx req; normalizes with bronchodilator; FEV1 or peak flow <70% of baseline | No Rx required; FEV1 or peak flow >80% of baseline |
| DYSPNEA | Dysnea on exertion | No dyspnea |

| **URINALYSIS** |  |  |
| --- | --- | --- |
| PROTEINURIA |  |  |
| Spot urine | 4+ | 2+ |
| 24 hour urine | > 1 g loss/day  **OR** > 1.0%  **OR** > 10 g/l | < 200 mg loss/day  **OR** <0.3%  **OR** <3 g/l |
| GROSS HEMATURIA | Gross hematuria | Microscopic hematuria only |

| **MISCELLANEOUS** | **HOLD IL-2 FOR:** | **CONSIDER RESTARTING**  **IL-2 FOR:** |
| --- | --- | --- |
| FEVER (oral) | >38.5^0^C **OR** 101.6^0^F for > 12 continuous hours  **OR**  40.5^0^C OR >105 for one reading | > 38.5^0^C **OR** 101.6^0^F intermittently only; no continuous fevers for >12 hours. |
| ALLERGIC REACTION | Generalized urticaria and/or angioedema | Pruritis without rash |
| CUTANEOUS/RASH/  DERMATITIS | Vesiculation **OR** moist desquamation **OR** ulceration | Ereythema and pruritis only |

**APPENDIX F. SYMPTOMATIC MEDICATIONS RECOMMENDED FOR MANAGEMENT OF IL-2 RELATED SIDE EFFECTS**

**OUTPATIENT MANAGEMENT GUIDELINES**

**SUPPORTIVE CARE**

GENERAL RECOMMENDATIONS:

1. Patients should receive a formal teaching session prior to outpatient treatment. In particular, they should be told that fever and malaise are to be expected and that nausea and anorexia are common. The importance of taking acetaminophen and a nonsteroidal antiinflammatory around the clock while awake should be emphasized. They should be educated regarding prn medications they can take without consultation and when they should consult a physician.

2. Patients receiving their treatment as outpatients should be closely monitored. Phone contact should occur daily and the patient should have 24-hour access to an experienced health care professional.

3. Patients must have a support system available to them during their treatment. A friend or family member should be available at the home in case of emergencies.

4 Patients should be encouraged to limit their normal activities during treatment since fatigue exacerbates most IL-2 symptoms.

5. In general, a diet of bland, soft foods eaten at frequent intervals is best tolerated. Adequate hydration is important and consumption of salty liquids such as canned soups and Gatorade is important to minimize hyponatremia.

6. Daily weights should be obtained on a reliable scale and should be monitored both for evidence of excessive fluid retention and inadequate volume intake. The patient should measure his oral input and urine output daily and record his temperature, pulse and respiratory rate three times a day while awake. Patients should gain about one pound/day.

**Concomitant medications (highly recommended to be given around the clock):**

1. Acetaminophen 650 mg p.o. 1 hour prior to beginning IL-2 and q4-6 hours around the clock while awake.

2. A nonsteroidal anti-inflammatory is strongly recommended. The NIAID uses ibuprofen 600 mg q6 hrs or Clinoril 150 mg q6 hrs alternating with acetaminophen. The NCI Surgery Branch uses indomethacin 50 mg p.o. q6 hrs around the clock while awake; this dose may be increased to 75 mg around the clock if the fever is inadequately controlled and if the patientÕs renal function is adequate.

**PRN Medications:**

1. Rash or itching: Atarax 25 mg t.i.d. or Benadryl 25-50 mg p.o. prn Eucerin cream or Nutraderm, Aveeno soap and baths are also helpful.

2. Severe chills/rigors: Demerol 25-75 mg p.o./IM q4-6 hrs prn.

3. Nausea or vomiting: Compazine 10 mg p.o. q4-6 hrs prn with lorazepam 1 mg p.o. q6 hrs. Compazine capsules 5-10 mg p.o. t.i.d. q8 hrs have also been used. Droperidol or ondansetron are more expensive alternatives.

4. For mouth ulcers: 2% solution of kaolin pectate, xylocaine and benadryl.

5. Diarrhea: codeine is the first line treatment at the Surgery Branch. This is followed by Immodium or Lomotil 2 tablets p.o. with each loose bowel movement (not to exceed 8/day). Tincture of opium has also been used in refractory patients.

6. Anxiety: Lorazepam 1 mg p.o. for anxiety. Dalmane or other mild sleeping pill is often helpful. These medications should be avoided in any patients with even subtle changes in their mental status.

7. Electrolytes: Mg^++^ supplementation and Ca^++^ supplements are often necessary and should be considered early in the outpatient setting. PO_4_ supplementation is occasionally necessary.

8. Dyspepsia: Cimetidine 300 mg p.o. q6 hrs or ranitidine 150 mg p.o. q12 hrs (probably not necessary for patients receiving the 3 day regimen).

**Signs or symptoms which require physician evaluation include (but are not limited to):**

- Urine output <500 cc/day
- Fever >102 which does not respond to antipyretics. Because IL-2 adds a neutrophil defect to the immune defect of the HIV seropositive patient, any suggestion of infection must be evaluated aggressively. Patients should have blood and other sites cultured as clinically indicated. It is important to note that the neutrophil defect associated with IL-2 treatment persists for at least one week following IL-2 completion.
- Pain or induration at subcutaneous injection site.
- Greater than mild anxiety or agitation; any signs of confusion, somnolence or disorientation; neurotoxicity from IL-2 is often progressive even when the IL-2 infusion has been discontinued.
- Respiratory rate of >25 or shortness of breath unassociated with fever
- Institutional norms should be considered in determining whether oral or intravenous supplementation of Ca^++^, Mg^++^, or PO_4_ is appropriate

**APPENDIX G. FLOW CYTOMETRY PANELS FOR IMMUNOPHENOTYPING -central laboratory**

**I. EQUIPMENT**

A. 12 x 75 mm polystyrene centrifuge tubes (Fisher, #B25754)

B. Eppendorf pipette (CMS, #109-967)

C. Pipette Tips (Fisher, #21-380-8B and #21-380-C)

D. Vortex

E. Centrifuge

F. Cotton Swabs

**II. REAGENTS**

A. Monoclonal Antibody Panel

ANTIBODY MANUFACT. CAT.# VOL/TEST

| **Limited Flow Panel (1-3)** | | | |  |
| --- | --- | --- | --- | --- |
| 1. IgG1 FITC x IgG2 PE | B-D^1^ | 335041 | 20 µL | |
| 2. CD3 FITC x CD4 PE | B-D | 335043 | 20 µL | |
| 3. CD3 FITC x CD8 PE | B-D | 349519 | 20 µL | |
|  |  |  |  | |

In addition, the following may be performed:

C.Neg.IgG1 FITC

C.Neg.IgG1 PE

CD45/CD14 (Leucogate)

CD3 PE

CD4 FITC

CD8 FITC

CD38 PE

CD45 RO Pe

CD45 RA FITC

For a total of 840 tests during a period of two years, 5 vials of each of the monoclonal antibodies are needed.

**APPENDIX H. PREPARATION GUIDELINES FOR HIV LOAD TESTING**

**bDNA SPECIMEN FOR HIV RNA**

**A. TYPE OF SPECIMEN**

1. 2ml sterile plasma (may need additional 2 ml specimen in case of retest).

**B. METHOD OF COLLECTION**

1. Blood should be collected in sterile K_3_ EDTA (lavender top) tubes.

2. Heparin (green top), ACD (yellow top), or Citrate will work but K_3_EDTA is better.

3. Use the same anti-coagulant throughout the study.

4. Whole blood should be stored at room temperature prior to separation of plasma from cells.

5. Separate plasma from cells as soon as possible (within 8 hours).

6. Store plasma in sterile screw-capped 1.5 ml Sarstedt conical tubes (catalog #72.692.005).

7. Store as 1 ml aliquots, accurately measured.

**C. STORAGE CONDITIONS**

1. Freeze 1 ml aliquots of plasma at -70¼C.

2. Avoid repeated thawing and refreezing.

**D. SHIPPING**

SHIPPING OF CLINICAL SAMPLES MUST BE IN COMPLIANCE WITH ALL APPLICABLE GOVERNMENT REGULATIONS AND CARRIED OUT BY APPROPRIATELY TRAINED INDIVIDUALS.

1. Ship on enough dry ice to last 72 hours.

**APPENDIX I. MODIFIED CPCRA ACCEPTABLE CRITERIA FOR CLINICAL HIV DISEASE PROGRESSION**

**1. Candidiasis, pulmonary**

Diagnosis is based on macroscopic inspection at endoscopy and histology of biopsy or cytology of specimen from the lung or a positive culture for candida species from pulmonary tissue taken at open lung biopsy.

Documentation: Clinical report and Histology report

**2. Candidiasis, esophageal**

Diagnosis is made by macroscopic inspection at endoscopy and histology of biopsy or cytology of specimen from mucosal surface (not by culture, not by barium swallow)

Documentation: Clinical report (including documentation of response to therapy).

Histology report or endoscopy report

**3. Cervical Cancer**

Invasive cervical cancer requires histological evidence obtained by cervical biopsy or at hysterectomy.

Cases of cervical dysplasia (pre-cancer or cervical intraepithelial neoplasia or squamous intraepithelial lesions) or cervical warts are not evidence of disease progression.

Documentation: Clinical report and Histology report

**4. Coccidioidomycosis, disseminated**

Diagnosis is based on microscopy (histology or cytology), culture or detection of antigen in a specimen obtained directly from the tissues affected or a fluid from those tissues.

Documentation: Clinical report and Microbiology report or Pathology report

**5. Cryptococcosis, systemic**

Diagnosis is based on clinical findings and microscopy or culture confirmation.

Documentation: Clinical report and Culture report or Microscopy report and Positive cryptococcal antigen.

**6. Cryptosporidiosis with diarrhea >1 month**

Diagnosis is based on stool microscopy in patients who have a history of persistent diarrhea (more than 3 stools per day) for more than one month. Other causes of chronic diarrhea are to be ruled out.

Documentation: Clinical report and Histology report or Stool microscopy report

**7. Cytomegalovirus disease, other than acute self-limited mononucleosis**

Diagnosis is assessed by the presence of consistent clinical findings associated with the presence of CMV inclusions in the tissue of the involved organ.

Documentation: Clinical report and Histology report and other relevant report (e.g. endoscopic report)

**8. Cytomegalovirus retinitis**

Diagnosis made by characteristic appearance on ophtalmoscopic examination. (Discrete patches of retinal whitening with distinct borders spreading in a centrifugal manner, frequently associated with retinal vasculitis, hemorrhage and necrosis).

Documentation: Clinical report and OphtalmologistÕs report

**9. Herpes simplex esophagitis**

Diagnosis is made by a positive HSV culture, microscopy or detection of antigen in a specimen obtained directly from affected tissue by endoscopy.

Documentation: Clinical report (including documentation of response to therapy) and Histology report or Culture report

**10. Herpes simplex pneumonitis**

Diagnosis made by a positive HSV culture, microscopy or detection of antigen in a specimen obtained directly from affected tissue by lung biopsy.

Documentation: Clinical report (including documentation of response to therapy) and Histology report or Culture report

**11. Herpes simplex bronchitis**

Diagnosis made by a positive HSV culture, microscopy or detection of antigen in a specimen obtained directly from affected tissue by bronchoscopy.

Documentation: Clinical report (including documentation of response to therapy) and Histology report or Culture report

**12. Herpes Zoster, disseminated**

Diagnosis made by biopsy with ten or more pocks occurrring outside the primary or adjacent dermatomes, or positive VZV viremia.

Documentation: Clinical report and blood culture or biopsy report.

**13. Histoplasmosis, systemic**

Diagnosis of histoplasmosis requires a positive *Histoplasma* culture from a non-pulmonary site or microscopic evidence (histology or cytology) of a non-pulmonary site or detection of antigen in a specimen obtained directly from the affected tissues or fluid.

Documentation: Clinical report and Culture report or Microscopy (histology or cytology) report

**14. Isosporiasis with diarrhea > 1 month**

Diagnosis is made by microscopy of the stool in a patient who must have a history of persistent diarrhea for >1month. Other causes of chronic diarrhea should be ruled out.

Documentation: Clinical report and Stool microscopy report

**15. KaposiÕs sarcoma with visceral involvement**

Confirmed diagnosis of visceral KaposiÕs sarcoma should be by biopsy and histological examination.

Documentation: Clinical report and Histology report

**16. Lymphoma (T cell, B cell or Hodgkin’s)**

Diagnosis is based on tissue biopsy or aspirate with histology demonstrating small non-cleaved cell lymphoma or a small lymphocytic type.

Documentation: Clinical report and Histology report

**17. Lymphoma of the brain**

Confirmed diagnosis proven by biopsy and histology**.**

Documentation: Clinical report and Histology report

Presumptive diagnosis may be based on the following:

1. Recent onset of a focal neurological abnormality consistent with intracranial disease or a reduced level of consciousness;

2. CT or MRI scan evidence of a lesion or lesions having a mass effect;

3. No response to standard toxoplasmosis treatment for a minimum of two weeks;

4. No evidence of lymphoma outside the brain.

Documentation: Clinical report and CT or MRI scan / report

**18. HIV-related encephalopathy or AIDS Dementia complex, stage 2 or higher**

Diagnosis is based on clinical findings of disabling cognitive and/or motor dysfunction (for at least one month) interfering with the patientÕs occupation or daily living activities. The diagnosis should include a Price and Brew staging system score >1.0.

AND

Methods to rule out concurrent disease: cerebrospinal fluid examination and MRI or CT scan.

Documentation: Clinical report and CSF report and CT or MRI scan report

**19. *M.avium* complex, systemic**

Diagnosis by culture of any sterile site. The patient must have a concurrent illness consistent with the diagnosis, e.g. weight loss, fever, diarrhea or anemia.

Documentation: Clinical report and Culture report

**20. *M.tuberculosis,* pulmonary or extrapulmonary**

Diagnosis by proven culture and imaging.

Documentation: Clinical report and CT or MRI scan/report

**21. *Mycobacterium,* other or unidentified species, systemic**

Diagnosis by culture from a site other than the lungs (including skin and cervical lymph nodes).

Documentation: Clinical report and Culture report

Presumptive diagnosis based on identification of acid-fast bacilli in stain of normally sterile site.

Documentation: Clinical report and acid-fast stain microscopy report

**22. *Penicillium marneffei*, disseminated**

A definitive diagnosis requires identification of the organism on biopsy or culture.

Documentation: Clinical report and Culture report or Histology report

**23. *Pneumocystis carinii* pneumonia, or extrapulmonaryPneumocystosis**

Diagnosis by cytology/microscopy or histology with sample collected via bronchoscopic biopsy or bronchoalveolar lavage or sputum.

Documentation: Clinical report and Histology report or Cytology report

A presumptive diagnosis can be based on the following criteria:

1. History of dyspnea on exertion, or non-productive cough (within 3 months),

2. If on PCP prophylaxis, typical CXR appearance of diffuse bilateral pulmonary infiltrate, or

3. If not on PCP prophylaxis for at least two weeks and CD4 count less than 200, CXR atypical or typical for PCP,

4. No evidence of bacterial pneumonia (documented by sputum and blood culture),

5. Response to PCP treatment.

Documentation: Clinical report and Chest X-ray report and Sputum culture report and Bronchoscopy report if performed and sequential LDH measurements if available.

**24. Progressive multifocal leukoencephalopathy**

A confirmed diagnosis of PML requires histological evidence obtained by brain tissue biopsy.

Documentation: Clinical report and Histology report

A presumptive diagnosis of PML can be based on progressive deterioration in neurological function together with MRI or CT scan evidence.

Documentation: Clinical report and MRI scan report and/or CT scan report

**25. *Salmonella* septicemia, non-typhoidal**

Diagnosis is based on blood culture .

Documentation: Clinical report and Blood culture report

**26. Toxoplasmosis of the brain ,or non-CNS**

A definitive diagnosis requires histology on a brain or other tissue biopsy.

Documentation: Clinical report and Brain biopsy histology report

A presumptive diagnosis of CNS toxoplasmosis is based on the following criteria:

a) Recent onset of a focal neurologic abnormality consistent with intracranial disease or a reduced level of consciousness;

AND

b) Evidence by brain imaging (computed tomography or nuclear magnetic resonance) of a lesion having mass effect or the radiographic appearance of which is enhanced by injection of contrast medium.

AND

c) Serum antibody to toxoplasmosis or successful response to therapy for toxoplasmosis

Documentation: Clinical report, CT/MRI report and serum antibody results

**27. Trypanosomiasis, American (Chagas Disease) of the CNS.**

A definitive diagnosis requires histology on a brain biopsy.

Documentation: Clinical report and brain biopsy histology report.

A presumptive diagnosis is based on the following criteria:

a) Evidence by brain imaging (computed tomography or nuclear magnetic resonance) of a lesion having mass effect that enhances with contrast and inadequate response to therapy for toxoplasmosis

AND

b) Serum antibodies to trypanosomiasis

AND

c) Peripheral blood smear or CSF smear positive for trypanosomal parasites or response to therapy for trypanosomiasis.

Documentation: Clinical report, CT/MRI report, parasitology and serum antibody results.

**APPENDIX J. 21 CFR 50 - REQUIREMENTS FOR INFORMED CONSENT**

**NIAID Informed Consent Guidelines**

This guide is a short summary of the elements required for all protocol consent forms. A more detailed discussion can be found in ÒProtomechanicsÓ, available from the NIH Clinical Center.

**Adult Consent (NIH 2514-1)**

Informed consent is a process, not just a form. Information must be presented to enable persons to decide whether or not to participate as a research subject, and in a manner that will minimize the possibility of coercion or undue influence. It is a fundamental mechanism to ensure respect for persons through provision of thoughtful consent for a voluntary act. The procedures used in obtaining informed consent should be designed to educate the subject population in terms they can understand. Therefore, informed consent language and its documentation must be written in Òlay languageÓ, interpretable by the people being asked to participate. No informed consent, whether oral or written, may include any language that appears to waive any of the subjectÕs legal rights, or releases the investigator, IND Sponsors or institution from liability or negligence.

Written presentation of information is used to document the basis for consent and for the subjectÕs future reference. The second person is generally used to address the subject. Use of the first person (e.g. ÒI understand thatÓ...) can be interpreted as suggestive, may be relied upon as a substitute for sufficient factual information, and can constitute coercive influence over a subject. Use of scientific jargon and legalese is not appropriate; lay equivalent suggestions are given as the ÒGlossaryÓ in the ÒLay Language Suggestions, Part AÓ , included in this packet. Think of the consent document primarily as a teaching tool, not as a copy of the protocol or a legal instrument.

**Purpose of the Study and Background Information**

Explain the general purpose of the study, including an invitation to participate and an explanation of why the subject was selected. Describe the overall experience that will be encountered, how the study is experimental, and how the subjectÕs involvement will develop the understanding of the disease process.

**Subject Population**

Summarize the inclusion and exclusion criteria in lay terms. If relevant, explain the need for and method of randomized assignment to treatment or placebo groups. The ÒLay Language SuggestionsÓ in Protomechanics Appendix I-1 and I-2 may be a useful aid to describing some of the research concepts.

Discuss any pre-study test results that must be obtained before actually starting the study, such as pregnancy and HIV testing. For female subjects capable of childbearing, the NIAID IRB requires a pregnancy test within two weeks prior to treatment and counseling concerning risks of pregnancy and effective contraception when appropriate.

**Procedures**

Describe the procedures that are to be performed, including a description of experimental versus non-experimental procedures and all research-related tests to be done. Dosing schedules and modifications based on side effects or abnormal laboratory results should be clearly discussed. See ÒDescriptions of Common Research ProceduresÓ in the ÒLay Language Suggestions, Part BÓ for help in wording. Explain how long the tests and procedures will take, their frequency, and where the procedures will be performed. Each procedure, when required as part of the study, must be described in the original consent form even if a separate consent form is to be signed later.

For blood drawing procedures, the volume of blood should be specified in lay terms such as teaspoons (one teaspoon is approximately equal to 5cc) or cupfuls. Although it is not necessary to include the following in the consent document, the NIAID IRB allows no more than 450 milliliters of blood per six week period from adults.

**Risks and Discomforts**

Inform the subject of the reasonably foreseeable harms, discomforts, inconvenience and risks that are associated with the research activity, and assess the probability of such a risk or discomfort. Possible complications of blood drawing include discomfort and occasional bruising at the site, and rarely fainting and local infection. One chest x-ray is considered acceptable. Additional chest x-rays must be medically indicated or reviewed by the Radiation Safety Committee.

Estimate the total amount of time required for participation. If additional risks are identified during the course of the research, the consent process and documentation will require revisions to inform subjects as they are recontacted or newly contacted.

**Benefits**

Describe the benefits that subjects may reasonably expect to encounter. There may be none other than a sense of helping the public at large. No claims should be made, either explicitly or implicitly, that the drug is safe or effective for the purposes under investigation, or that the drug is in any way equivalent or superior to any other drug. Such representation would not only be misleading to subjects but would also be a violation of the FDAÕs regulations concerning the promotion of investigational drugs (21 CFR 312.7 (a)).

**Compensation**

Payment of research subjects for participation in studies is considered a compensation. If payment is given to defray the incurred expense for participation or potential discomfort, it must not be coercive in amount or method of distribution. All information concerning payment, including the amount and schedule of payment, must be set forth in the informed consent form.

**Alternative Therapy**

Describe any alternatives to participating in the research project. For example, in drug studies the medication(s) may be available through their family doctor or clinic without the need to volunteer for the research activity, or other regimens may treat the condition as well.

**New Findings**

Describe the process that will ensure the subject that pertinent new findings in the literature or findings discovered during the project will be fully discussed.

**Contacts**

The Principal Investigator and any Associate Investigators available for contact should be listed on the final page of the consent form. Study participants must have ready access to the personnel involved in the study to ask questions and report their condition or any problems.

**INCLUSION OF WOMEN AND MINORITIES**

Regarding IRB implementation, the new Guidelines on the inclusion of Women and Minorities in Clinical Research have been discussed with the NIH Human Subjects Research Advisory Committee (HSRAC), which includes all the NIH IRB Chairs, and with the Clinical Center Medical Board. Effective July 15, 1994, the following requirements will apply to all new research protocols submitted to NIH IRBÕs for review:

1. Design of protocols: In their clinical research protocols, PIs must include:

(a) the rationale for the research subject selection based on a review of the gender and population category(ies) at risk for the disease or condition being studied;

(b) strategies and procedures for recruiting the subject population selected in (a), above, and

(c) justification for exclusions, if any, of women and/or individuals from particular population categories.

2. Initial IRB review of protocols: The IRB is required to review and approve the rationale for research subject selection; the strategies and procedures for recruiting subjects, and the justification for exclusion of women and/or individuals from particular population categories. Exclusions may be warranted because of the nature of the disease or condition being studied or there may be other justifiable reasons.

3. Continuing IRB review of protocols: After July 15, 1994, continuing review and approval of clinical research protocols approved by IRBs must include a review of subject accrual by gender and ethnic/racial category. Until the Continuing Review Application form NIH 1195-1 is amended, PIs must include in the memorandum which accompanies their continuing review application to the IRB the cumulative number of subjects enrolled, broken down by gender and racial/ethnic population category(ies).

**¥ The NIH Guidelines identify the following population categories: American Indian or Alaskan Native, Asian or Pacific Islander, Black, Hispanic, and White (see page 13 of the Guidelines). To the extent possible, identification of subject population(s) should be consistent with these categories.**

**ISSUES TO BE INCORPORATED IN INFORMED CONSENT WHEN GENETIC TESTING WILL BE PERFORMED.**

(Not to be considered *boiler plate*  language.)

In addition to the actual research protocol, there are several general points that we want to mention to help you understand issues that are indirectly related to your participation in the research study:

**1.** **Future research:**

Our specific research plans have been summarized in this consent form. In the course of the research, we will obtain blood samples. While we can do some of our tests immediately, we may store your blood samples in the freezer for future studies. In addition, certain information will be stored in your medical record. In the future, it is possible that your blood samples or your medical records will be used for other research purposes that are not specifically outlined in this consent form. If this is done, the samples will be coded, but you will not be identified by name. In addition, it is possible that some information obtained from these studies will be published in the medical literature. However, your identity will not be included in these publications.

**2.** **Unanticipated medical information:**

During the course of this investigation, it is possible (although not likely) that we will obtain unanticipated information about your health or genetic background. If this information is considered to be relevant to your health care, we will provide it either to you or to your referring physician.

**3.** **Release of medical records:**

In the course of applying for certain types of insurance (e.g., medical insurance, life insurance, or disability insurance), people are often asked to sign forms that authorize insurance companies to obtain their medical records. If you sign such a release form at some point in the future, it is possible that the insurance company would present this signed release form to the Clinical Center of the National Institutes of Health. In that event, the National Institutes of Health would comply with your request to provide the insurance company with your medical record. It is possible that the information contained in your medical record might affect (either favorably or unfavorably) the willingness of the insurance company to sell you insurance.

**4.** **Family relationships:**

In the course of this study, it is possible that we may learn information about relationships within the family. For example, it is possible that we might learn that a family member is not the biological child of the parents with whom he/she lives (for example, because of adoption). We will not ordinarily provide this type of information to any member of the family or the referring physician. However, we may make exceptions under extraordinary circumstances if this information were required for the medical care of the individuals involved. If we are convinced this is necessary, we will provide the information to the physician providing medical care to the patient.

**5.** **Participation in other research studies:**

This consent form specifically refers to your participation in the research protocol described above. In the future, it is possible that we may invite you to participate in other studies. Even if you sign this consent form, you are not obligated to participate in these other research protocols. If you are asked to participate in other research protocols, you will be provided with additional consent forms. As stated in the introduction to this protocol, you are free to withdraw from any or all research studies at any time without penalty or loss of any benefits to which you are otherwise entitled.

**6.** **Genetic Counseling:**

Counseling will be provided to you when you receive the results of your test. If you wish, counseling will be available to you before you agree to undergo testing.

**APPENDIX K. PRICE AND BREW STAGING SYSTEM FOR THE AIDS DEMENTIA COMPLEX**

**Staging scheme for the AIDS dementia complex (ADC)***

**ADC stage Characteristics**

Stage 0 Normal mental and motor function

(Normal)

Stage 0.5 Either minimal or equivocal symptoms of (equivocal/subclinical) or cognitive motor dysfunction characteristic of ADC, or mild signs (snout response, slowed extremity movements), but *without impairment of work or capacity to perform activities of daily living* (ADL); gait and strength are normal.

Stage 1 Unequivocal evidence (symptoms, signs,

neuropsychological test performance) of functional, intellectual or motor impairment characteristic of ADC, but able to perform *all* *but the more demanding aspects of work or;* can walk without assistance.

Stage 2 Cannot work or maintain the more

demanding aspects of daily life, but able to perform *basic activities of self-care*; ambulatory, but may require a single prop.

Stage 3 *Major intellectual incapacit*(cannot follow news or personal events, cannot sustain complex conversation, considerable slowing of all output), *or motor disability* (cannot walk unassisted, requiring walker or personal support, usually with slowing and clumsiness of arms as well).

Stage 4 *Nearly vegetative* ; intellectual and social comprehension and responses are at a rudimentary level; nearly or absolutely mute; paraplegic with double incontinence.

*The ADC stage is applied only after establishing a clinical diagnosis of ADC, which requires the presence of documented HIV-1 infection, evidence of an *acquired*  neurologic deficit, and exclusion of other neurologic or psychiatric disorders explaining this deficit. It also requires the presence of characteristic clinical abnormalities in cognitive, motor, or behavioral functions. In the cognitive sphere this may include early symptoms of impaired concentration and slowed thinking. Complaints of forgetfulness are common, but early in ADC formal memory testing may reveal normal function. Cognitive changes may then evolve to significant impairments in abstraction, profound slowing, failures in concentration and memory, and later, to global dementia. In the motor sphere, characteristic ADC changes include early slowing of rapid extremity movements, which may evolve to clumsiness and ataxia of gait and impaired hand coordination, and later to paraplegia, with incontinence in the most severe cases. Behavioral dysfunction has somewhat less diagnostic precision, but characteristic abnormalities include early personality change and apathy without dysphoria, which may progress hand in hand with the psychomotor retardation to the point of near or even frank mania. Laboratory studies of ancillary value include neuro-psychological assessment in which the characteristic profile demonstrates psychomotor slowing and poor performance on tasks of attention and concentration, with relative sparing of language and memory skills early in the course; CT or MRI showing brain atrophy and, in some, white matter or basal ganglion abnormalities; and elevated CSF beta_2_ - microglobulin or neopterin in the absence of other causes.
